# Exhaustion disorder: scoping review of research on a recently introduced stress-related diagnosis

**DOI:** 10.1192/bjo.2022.559

**Published:** 2022-08-24

**Authors:** Elin Lindsäter, Frank Svärdman, John Wallert, Ekaterina Ivanova, Anna Söderholm, Robin Fondberg, Gustav Nilsonne, Simon Cervenka, Mats Lekander, Christian Rück

**Affiliations:** Center for Psychiatry Research, Department of Clinical Neuroscience, Karolinska Institutet, Stockholm, Sweden; Stockholm Health Care Services, Stockholm, Sweden; and Division of Psychology, Department of Clinical Neuroscience, Karolinska Institutet, Stockholm, Sweden; Center for Psychiatry Research, Department of Clinical Neuroscience, Karolinska Institutet, Stockholm, Sweden; and Stockholm Health Care Services, Stockholm, Sweden; Department of Psychology, Umeå Universitet, Umeå, Sweden; Division of Psychology, Department of Clinical Neuroscience, Karolinska Institutet, Stockholm, Sweden; and Stress Research Institute, Department of Psychology, Stockholm University, Stockholm, Sweden; Center for Psychiatry Research, Department of Clinical Neuroscience, Karolinska Institutet, Stockholm, Sweden; Stockholm Health Care Services, Stockholm, Sweden; and Department of Medical Sciences, Psychiatry, Uppsala University, Uppsala, Sweden; Division of Psychology, Department of Clinical Neuroscience, Karolinska Institutet, Stockholm, Sweden; Stress Research Institute, Department of Psychology, Stockholm University, Sweden; and Osher Center for Integrative Medicine, Department of Clinical Neuroscience, Karolinska Institutet, Stockholm, Sweden

**Keywords:** Review, psychological stress, fatigue, burnout, nosology

## Abstract

**Background:**

Symptoms related to chronic stress are prevalent and entail high societal costs, yet there is a lack of international consensus regarding diagnostics and treatment. A new stress-related diagnosis, exhaustion disorder, was introduced into the Swedish version of ICD-10 in 2005. Since then, use of the diagnosis has increased rapidly.

**Aims:**

To create the first comprehensive synthesis of research on exhaustion disorder to report on the current state of knowledge. Preregistration: Open Science Framework (osf.io), doi 10.17605/OSF.IO/VFDKW.

**Method:**

A PRISMA-guided scoping review of all empirical studies of exhaustion disorder was conducted. Searches were run in the MEDLINE, PsycInfo and Web of Science databases. Data were systematically charted and thematically categorised based on primary area of investigation.

**Results:**

Eighty-nine included studies were sorted into six themes relating to lived experience of exhaustion disorder (*n* = 9), symptom presentation and course (*n* = 13), cognitive functioning (*n* = 10), biological measures (*n* = 24), symptom measurement scales (*n* = 4) and treatment (*n* = 29). Several studies indicated that individuals with exhaustion disorder experience a range of psychiatric and somatic symptoms beyond fatigue, but robust findings within most thematic categories were scarce. The limited number of studies, lack of replication of findings and methodological limitations (e.g. small samples and scarcity of specified primary outcomes) preclude firm conclusions about the diagnostic construct.

**Conclusions:**

More research is needed to build a solid knowledge base for exhaustion disorder. International collaboration regarding the conceptualisation of chronic stress and fatigue is warranted to accelerate the growth of evidence.

Chronic stress, i.e. prolonged psychophysiological activation in response to perceived threat or challenge, is associated with symptoms of fatigue, disturbed sleep and cognitive deficits, as well as with an increased risk for adverse health outcomes across a range of medical disorders.^1,2^ Total societal costs for work-related stress in the Western world, mainly attributable to productivity loss and sickness absence, have been estimated at US$187 billion.^3^ Despite the significant burden for afflicted individuals and society at large, there is still no international consensus regarding how chronic stress-related symptoms induced by work or other environmental factors should be diagnosed or treated.^4^

Following the rise in sickness absence in Sweden in the late 1990s, a group of researchers investigated the development. After an interview study, to this day unpublished, with individuals on sick leave for depression, the researchers concluded that the clinical picture in a subgroup of individuals differed from depression, showing symptoms of prolonged physical and emotional fatigue that they commonly attributed to work-related stress. The emphasis on fatigue was considered to not be sufficiently covered by adjustment disorder, an already established stress-related diagnosis, and the researchers proposed provisional criteria for ‘exhaustion disorder’ (see the Appendix below for diagnostic criteria).^5^ Exhaustion disorder, conceptually similar to the burnout construct,^6^ was accepted into the Swedish version of ICD-10 in 2005 as a specification of F43.8 Reaction to severe stress, given the diagnostic code F43.8A.

In the original report presenting diagnostic criteria for exhaustion disorder,^5^ there was a strong emphasis on the need for research to validate the new diagnosis, including studies of aetiology, predisposing factors, pathophysiology and clinical factors related to measures, treatment and rehabilitation strategies. To date, the progress of this field has not been comprehensively assessed. Since the introduction of exhaustion disorder, however, the diagnosis has become almost as prevalent as major depression in Swedish healthcare settings^7^ and currently accounts for more instances of long-term sick-leave reimbursement than any other single diagnosis in the country.^8^ Notably, despite the alleged resemblance to internationally recognised concepts such as burnout^6^ and fatigue,^9^ the diagnosis of exhaustion disorder has not been included in any ICD-10 version outside of Sweden or in ICD-11.

## Aim of the review

The present review aimed to systematically chart all empirical studies that have investigated any aspect of exhaustion disorder, with the purpose of both reporting the present state of knowledge on the disorder and identifying knowledge gaps.

## Method

The registration and full protocol for the present review are available at Open Science Framework (osf.io) under the doi 10.17605/OSF.IO/VFDKW. Guidelines by Levac et al^10^ on extracting and reporting data for scoping reviews were followed. The Preferred Reporting Items for Systematic Reviews and Meta-Analysis extension for Scoping Reviews (PRISMA-ScR) checklist was used to guide reporting.^11^

### Eligibility criteria

All original empirical studies on individuals with an exhaustion disorder diagnosis were eligible for inclusion. Because exhaustion disorder (*utmattningssyndrom*) is a Swedish diagnosis with no established English translation, the terms ‘stress’, ‘burnout’, ‘adjustment disorder’ and ‘exhaustion’ were included in the literature searches (supplementary Table S1, available at https://doi.org/10.1192/bjo.2022.559), and studies referring to specific exhaustion disorder diagnostic criteria in the recruitment and inclusion procedures were identified. Both qualitative and quantitative studies were eligible, as were studies including other diagnoses in addition to exhaustion disorder if the proportion of participants with exhaustion disorder could be identified. For diagnostic distinction purposes, studies lacking clear reference to exhaustion disorder diagnostic criteria were excluded, even when those studies focused on similar concepts (e.g. ‘burnout’).

### Search strategy

Articles were searched in the MEDLINE, PsycInfo and Web of Science databases. The search strategy was developed in MEDLINE and then adapted to the syntax of the other databases in collaboration with a research librarian at the Karolinska Institutet University Library. No restrictions were imposed on the searches regarding publication year, publication status or language. Further, ClinicalTrials.gov and PROSPERO were searched to identify completed trials and relevant reviews respectively. Reviews were not included but their reference lists were screened for potentially missed references. Information not formally published as articles in scholarly journals was searched in publications from the Swedish National Board of Health and Welfare and other public authorities, as well as in practice-oriented journals and Swedish university dissertations. The first search was conducted on 17 June 2020. The database search was rerun before final compilation of results on 7 June 2021, to include recently published findings. Rayyan (https://rayyan.qcri.org/), a web-based tool for screening and selecting studies in systematic reviews, was used to aid in the assessment of inclusion/exclusion.

### Selection of sources of evidence

Titles and abstracts of all publications were screened independently and in parallel by two reviewers (F.S. and V.L.). In a second phase, full articles were divided between two reviewers (E.L. and F.S.) and screened for final inclusion. Reviewers conducted a calibration exercise at the beginning of each phase which included reviewing the 20 first articles and checking for coherence. In cases of uncertainty or disagreement between reviewers, the larger research group was consulted, and a consensus decision was required for inclusion.

For the purpose of the present study, studies in which reference to diagnostic criteria for exhaustion disorder was not clear in the described recruitment and/or assessment procedures, in which the number of individuals diagnosed with exhaustion disorder could not be deciphered or in which exhaustion disorder was identified solely on the basis of a self-report questionnaire were excluded from the review. Over 25 investigators were contacted for clarifications about sampling and diagnostic procedures. Supplementary Table S2 presents a non-exhaustive overview of excluded but ‘complementary’ studies in which it was unclear to what extent study participants met diagnostic criteria for exhaustion disorder, but where samples possibly overlap with exhaustion disorder samples.

### Data-charting process

A data-charting form was produced *a priori* and used for included studies. This included (a) first author, (b) year of publication, (c) study location, (d) study aims, (e) design, (f) sample/recruitment strategy, (g) sample size, (h) methodology, (i) primary outcomes and (j) main findings. Because the review is characterised as scoping and consequently aims to include all types of study, no predefined outcomes were selected and no formal risk of bias assessment of individual studies was conducted. Of note, because primary outcome measures were not always stated and several studies reported a range of non-hierarchical findings, the research group selected findings that were perceived as most central to the study aim. The reader is referred to the respective publications for comprehensive accounts of results. To specifically inform clinicians, a more comprehensive data-charting form for primary quantitative studies evaluating the effect of exhaustion disorder interventions was constructed (including, for example, follow-ups and attrition) and is presented in supplementary Tables S3a and S3b.

### Synthesis of results

Studies were organised into thematic categories based on their primary investigation area. Because included studies varied significantly in design and ranged from experimental physiological studies to qualitative studies using a phenomenological approach, specialists within each area of the different methodological approaches were included in the research team. To restrict bias in the interpretation of methods and findings, the specialists were chosen from both within and outside of the exhaustion disorder research field. Recurring meetings and workshops were arranged for the research team to discuss findings and generate syntheses in each thematic category, with a focus on describing the aims, methodology and main findings of studies in respective areas.

## Results

### Search results

**Fig. 1:**
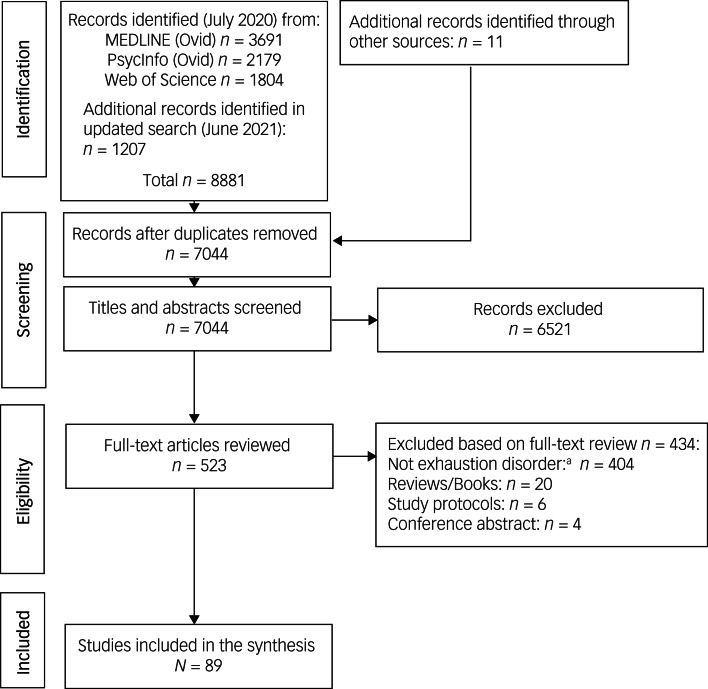
PRISMA flow diagram illustrating the inclusion and exclusion process for the review. a. Includes the 39 studies that were excluded owing to unclear reference to exhaustion disorder diagnostic criteria or self-rated exhaustion disorder.

The inclusion process is depicted in Fig. 1. After screening, a final sample of 89 studies remained (supplementary Table S4). Thirty-nine studies with unclear definition of the exhaustion disorder diagnosis, unclear number of participants with the disorder or with self-rated exhaustion disorder were excluded but are presented as ‘Complementary studies’ in supplementary Table S2. Seventeen university dissertations were found that included empirical investigations of exhaustion disorder, but these generated no studies that had not already been reviewed for inclusion. Supplementary Table S5 presents an overview of the dissertations.

### Study characteristics

**Fig. 2:**
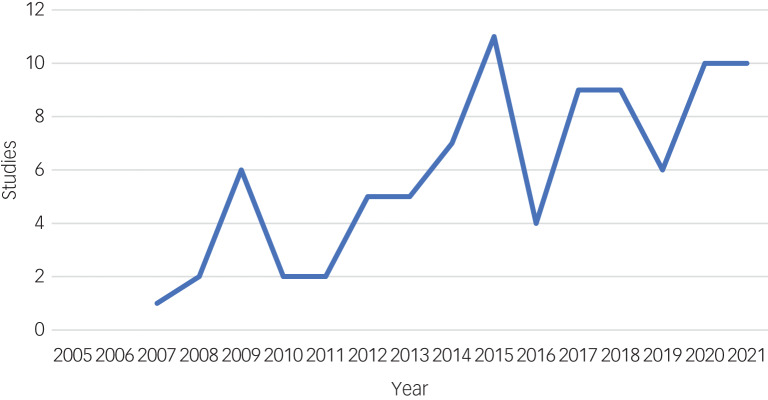
Number of included empirical studies of exhaustion disorder (Swedish ICD-10 F43.8A) (*n* = 89) presented by year of publication.

Figure 2 shows the number of included studies per publication year. Three studies were conducted in Norway and the remainder in Sweden. Fifty-six studies (63%) recruited exhaustion disorder samples from specialised clinics focusing on stress rehabilitation, 12 (13%) used samples from primary care, 10 (11%) recruited via the social insurance agency or private insurance companies, 6 (7%) via advertisement and 5 (6%) gave no information about sample recruitment. Supplementary Table S4 gives an overview of included studies, including study designs, sample sizes and the proportion of women in each study. To summarise, most studies used cross-sectional designs (*n* = 43; 48%), of which 23 (26%) were controlled and 13 (15%) were qualitative interview studies. Thirty-eight studies (43%) applied repeated/longitudinal measures, of which 12 (13%) were uncontrolled cohort studies, 9 (10%) used non-randomised controlled designs, 8 (9%) were randomised controlled trials (RCTs), 8 (9%) were secondary/subgroup analyses based on 4 of the original RCTs, and 1 was a longitudinal qualitative study. Of the remaining studies, three applied both cross-sectional and longitudinal designs and three used mixed methods. Two studies were observational medical chart studies.

### Synthesis of results

Based on the primary aim of each included study, six overarching thematic categories were constructed: lived experience of exhaustion disorder; symptoms, course and context; cognitive functioning; biological measures; symptom measurement scales; and interventions (supplementary Table S4). Studies that investigated aspects relevant to more than one theme (e.g. intervention and biological measures) are presented in more than one category below.

#### Lived experience of exhaustion disorder

Of the nine studies that qualitatively investigated lived experience of exhaustion disorder, six were conducted in Sweden and three were conducted in Norway using Norwegian participants who were diagnosed according to exhaustion disorder diagnostic criteria.^14–16^ One study interviewed co-workers of individuals with exhaustion disorder using thematic analysis;^17^ the other studies investigated exhaustion disorder samples, four using a phenomenological approach,^14–16,18^ three using a hermeneutic phenomenological approach^12,13,19^ and one using grounded theory.^20^ All of these studies collected data through face-to-face interviews, with some complementary sources: diaries,^14–16^ photographs,^18^ telephone interviews and email dialogues.^13^

In two studies, participants with exhaustion disorder described a starting phase where the body was perceived as recurrently calling for attention through a range of physiological symptoms (e.g. high blood pressure, dizziness, bodily weakness and disturbed sleep).^13,19^ Some participants described a struggled to keep working and manage everyday life despite feeling ill.^13,19^ In studies by Jingrot & Rosberg^19^ and Alsén et al,^12^ participants described a gradual loss of self-recognition related to an inability to engage in activities and feel joy like before. Feelings of shame, frustration and detachment from the body and the world were described^12,14–16,19^ and were perpetuated by the perception of not being listened to.^14,19^ In a study by Ericson-Lidman & Strandberg,^17^ co-workers of individuals with exhaustion disorder described that their afflicted colleagues became increasingly distanced and withdrew from others. Participants in two studies^14,15^ from Norway, where exhaustion disorder is not an established diagnosis, described struggling in contacts with healthcare providers because of symptoms being medically unexplained and uncertainty of the diagnosis.

After an extended period of ‘fighting for survival’,^19^ participants in some of the studies described a breakdown, or collapse, at which point they realised that they were very ill and ceased to struggle.^13,19^ Co-workers of individuals with exhaustion disorder described similar observations of their workmates ‘falling apart’.^17^ This collapse was also described as a ‘cross-roads’ for identifying new personal values and/or a new direction in life.^12,13,19^

To recover from exhaustion disorder, some participants described a period relieved of demands as essential.^13,18^ Aspects such as balancing energy,^13,16,18^ performing self-chosen creative activities,^13^ having joyful moments^16^ and a supportive environment^13,18,20^ were also described as important to aid recovery. Further, acquiring adaptive coping skills, gaining insights and being open to new possibilities were described as important in the rehabilitation process.^16,20^

##### Summary and critical appraisal

The studies provide descriptions of living with exhaustion disorder that highlight physiological symptoms, loss of self-recognition, struggle, withdrawal, detachment and breakdown, as well as aspects perceived to be important for recovery. Although results provide a deeper understanding of the lived experience of exhaustion disorder, sample sizes (*N*s = 8–18) are smaller than in the quantitative studies and results are not generalisable to other contexts. The studies from Norway had the same eight participants in all three studies.

#### Symptoms, course and context

Of the 13 studies included in this category, seven were cross-sectional and described self-rated symptoms and experiences.^22,26–29,31,32^ Four longitudinal cohort studies explored within-group symptom progression over time,^23–25,33^ of which one was based on medical chart data.^33^ Another two studies investigated symptoms^21^ and stressors^30^ as reported in medical charts for individuals diagnosed with exhaustion disorder.

##### Symptoms

Glise et al^24^ reported that on a checklist of 12 somatic symptoms, 45% of participants with exhaustion disorder reported six or more symptoms. Problems with flatulence or indigestion were most common (67%), followed by headaches (65%) and dizziness (57%). In the same study, 98% reported that they felt tired or had low energy, and 85% reported disturbed sleep.^24^ A retrospective medical chart review by Adamsson & Bernhardsson^21^ showed that in the 2 years preceding an exhaustion disorder diagnosis, individuals visited a general practitioner an average of 5.2 times (s.d. = 3.7), most often with complaints of infection, anxiety/depression and gastrointestinal symptoms. Medical charts revealed that 53% were diagnosed with a comorbid mental disorder and 61% with a comorbid somatic disorder.^21^ Ristiniemi et al^91^ (results further reported in the Interventions section below) found that self-rated hyperventilation was more common in individuals with exhaustion disorder than in healthy controls.

In a sample of 420 individuals with exhaustion disorder, Grossi et al^27^ found that more than 9 h of sleep per night (reported by 14% of participants) was associated with higher self-rated sleep quality but also with higher frequency of sick leave, depression, fatigue and daytime sleepiness compared with those who slept less than 9 h per night. In another study by Grossi et al,^28^ self-reported hazardous drinking was found to occur in 12% of women and 13% of men with exhaustion disorder. In women only, hazardous drinking was associated with lower mental well-being, but the subgroup of non-drinking women reported poorer physical health and more pain.^28^ Maroti et al^31^ found individuals with exhaustion disorder to self-report higher levels of anxiety, depression and difficulties identifying and describing feelings compared with individuals diagnosed with chronic fatigue syndrome and healthy controls, but exhaustion disorder participants did not differ from comparison groups on an observer-rated emotional awareness test. In studies by Maroti et al^32^ and Grensman et al,^26^ lower quality of life was reported in individuals with exhaustion disorder compared with healthy controls.

##### Symptom course and contextual factors

In a longitudinal cohort study by Glise et al,^23^ the course of symptoms of participants with exhaustion disorder in multimodal rehabilitation was followed over a period of 18 months (effects of the intervention were not formally assessed). The proportion of individuals who scored ≥4 points on the Shirom–Melamed Burnout Questionnaire (SMBQ), indicative of clinical burnout, was significantly reduced from baseline to 18-month follow-up, as was the proportion who reached clinical levels of depression and anxiety.^23^ Similarly, in the same cohort, most self-reported somatic symptoms decreased from baseline to the 18-month follow-up.^24^ A follow-up after 7–10 years of largely the same cohort indicated that almost half of individuals who were diagnosed with exhaustion disorder at baseline still reported fatigue, and the most common residual symptom was reduced stress tolerance (reported by 73%).^25^ Clinical assessment of a subgroup (*n* = 163 of 217) of participants indicated that one-third were still clinically exhausted (defined as either fulfilling diagnostic criteria or scoring ≥4.4 on the SMBQ with physician-confirmed residual symptoms of exhaustion disorder). However, 87% of participants in the total investigated sample were no longer on sick leave at follow-up.^25^ Cross-sectional studies of individuals in largely the same cohort, 7–10 years after enrolling in treatment for exhaustion disorder, indicated that most participants (67%) had made changes at work since treatment start (regarding workplace, work tasks or working hours), and women were more likely than men to have made changes in working hours.^22^ Those who had not recovered from exhaustion disorder reported higher incidence of obsessive–compulsive personality disorder compared with those who had recovered.^29^ Two studies report that quantitative work demands were the most prevalent stressors for individuals with exhaustion disorder,^26,30^ followed by private relational conflicts.^30^ In a prospective medical chart study, Skoglund et al^33^ investigated whether antidepressant medication was associated with sick leave during a 12-month period but found no such association.

##### Summary and critical appraisal

A range of psychiatric and somatic symptoms have been reported in individuals with exhaustion disorder. Although symptoms tend to decrease over time, some studies report residual symptoms up to 10 years after participants sought treatment. Conclusions regarding exhaustion disorder-specific symptom presentation and course are limited by the relatively small number of studies and by the lack of both population-based control groups and comparisons with other diagnostic groups. Further, exhaustion disorder participants included in studies often met criteria for concurrent depression and/or anxiety disorders, which complicates conclusions regarding disorder-specific symptoms and course. Many studies (6 out of 13) were conducted in the same cohort from a specialised treatment centre, which restricts generalisability of findings to other contexts and to more recently diagnosed individuals.

#### Cognitive functioning

Ten studies investigated cognitive functioning in individuals with exhaustion disorder. Of these, eight used cross-sectional designs, of which five compared cognitive test results of exhaustion disorder participants with healthy controls,^35,37,39–41^ one compared results of exhaustion disorder participants with normative test scores,^34^ one compared cognitive test results in former exhaustion disorder individuals with healthy controls^43^ and one compared subjectively self-assessed cognitive impairment (no cognitive testing) between individuals with exhaustion disorder, former exhaustion disorder and healthy controls.^36^ The two remaining studies investigated within-participant change in cognitive functioning in exhaustion disorder cohorts, with follow-up assessments after approximately 1.5^42^ and 3^38^ years. The latter study was coupled with a cross-sectional comparison of cognitive test results in former exhaustion disorder individuals and healthy controls at the 3-year follow-up.^38^

In addition to the above-mentioned studies, three studies based on an RCT investigated the effect of physical exercise^74^ and cognitive training^86^ on cognitive functioning in exhaustion disorder participants using a cognitive test battery, as well as long-term effects of the interventions.^87^ These studies are reported in the Interventions section below.

##### Cross-sectional cognitive testing

Österberg et al^41^ found that participants with exhaustion disorder performed significantly worse than healthy controls on a test of psychomotor speed, but not on tests measuring attention, working memory, episodic memory and vocabulary. In contrast, Jonsdottir et al^37^ found that exhaustion disorder participants performed significantly worse with respect to working memory, episodic memory and on one but not another test of executive function compared with controls. The study, however, found no differences between exhaustion disorder and control participants on the majority of administered cognitive tests. A study by Krabbe et al,^39^ with a particular focus on executive functioning and attention, compared individuals with exhaustion disorder with matched healthy controls and found that the exhaustion disorder group performed worse on a test of sustained attention as well as on a test of verbal fluency, but no differences were found regarding working memory. In a study by Ellbin et al,^35^ individuals with exhaustion disorder performed worse on tests of speed and attention, language and executive functioning relative to healthy controls, but not on immediate and delayed recall (memory) or on performance on a visuo-spatial task. Bartfai and colleagues^34^ investigated whether a tablet-based serial naming task could identify cognitive impairment in exhaustion disorder participants relative to normative data. The study also administered a cognitive assessment battery tapping working memory, general intelligence, information processing/perception, psychomotor speed and attention. Individuals with exhaustion disorder performed within population test norms on all tests except for the serial naming task.^34^ A study by Nelson et al^40^ compared a host of cognitive test outcomes in individuals with exhaustion disorder with those of matched controls and found mild cognitive impairment in exhaustion disorder on some tests of executive function and general intelligence, but no group differences for the bulk of assessed cognitive domains.

##### Follow-up of cognitive functioning

Österberg et al^42^ followed up former exhaustion disorder participants 1.5 years after initial sick leave (11% still fulfilling diagnostic criteria) and found improvements in working memory, processing speed, attention and episodic memory relative to baseline. When cross-sectionally comparing largely the same sample with a healthy control group 2 years after initial sick leave, participants with former exhaustion disorder (13% fulfilling diagnostic criteria) performed worse than controls on a continuous performance test and slightly worse regarding visuo-constructive ability, but outperformed controls on some tests of working memory, episodic memory and cognitive control.^43^ On other tests there were no differences between groups. In contrast, when Jonsdottir and colleagues^38^ conducted a follow-up of exhaustion disorder participants after an average of 3 years (23% still fulfilling diagnostic criteria), they performed worse than healthy controls regarding episodic memory and on some tests of working memory and language. In that study, within-group analyses showed no major changes in cognitive functioning among exhaustion disorder participants across time, even though substantial recovery from the disorder had taken place.^38^

##### Self-reported cognitive impairment

As regards subjective (as opposed to psychometrically tested) cognitive impairment, effect sizes were large and consistent across studies in domains of attention^41,42^ and memory,^35,37,39–42^ test-related fatigue,^39^ combinations of memory and attention/concentration^39,40^ and global cognition.^40^ In most studies there was a lack of coherence between subjectively reported cognitive impairment and tested cognitive performance. A notable exception is from Ellbin et al,^35^ reporting that self-assessed memory problems were associated with worse scores on tests of both immediate and delayed recall. Ellbin et al^36^ also found that exhaustion disorder participants who still fulfilled diagnostic criteria 7–10 years after study inclusion reported more subjective cognitive impairment compared with those who had recovered, who in turn reported more cognitive impairment than healthy controls.

##### Summary and critical appraisal

In summary, psychometric testing of cognitive functioning in individuals diagnosed with exhaustion disorder indicate, at most, mild cognitive impairment. However, results regarding the implicated domains are inconsistent across studies. Contrastingly, results for individuals with exhaustion disorder display large and consistent effect sizes for subjective self-reported cognitive impairment compared with controls. In general, the reviewed studies employed well-designed neuropsychological testing procedures and administered established and validated psychometric tests. However, sample sizes were often limited, and most studies investigated a range of cognitive outcome measurements across several domains without a clearly defined primary outcome, leading to an increased risk of both type I and type II errors. Moreover, the vast majority of studies did not employ proper population sampling of controls, which introduces a risk of sampling bias and makes between-group comparisons difficult to interpret.

#### Biological measures

Twenty-four studies had a primary aim of investigating biological aspects of exhaustion disorder, including the hypothalamus–pituitary–adrenal (HPA) axis (*n* = 5),^47,49,56,59,61^ the adrenal hormone dehydroepiandrosterone sulfate (DHEA-s) (*n* = 3),^48,50,51^ growth factors and related molecules in plasma (*n* = 6),^45,46,62,65–67^ structural and functional brain imaging measures (*n* = 5)^54,55,57,58,63^ and various other biological measures (*n* = 5).^44,52,53,60,64^ In addition to the 24 studies in this synthesis, 2 studies that primarily aimed to investigate cognitive functioning in exhaustion disorder participants^41,42^ and 1 RCT of treatment effect^90^ also reported HPA-axis measures. Of the 24 studies, 13 used a cross-sectional design and 11 used repeated measures.

##### Hypothalamus–pituitary–adrenal axis and adrenal hormone studies

Of studies that investigated the cortisol awakening response in individuals with exhaustion disorder, one study by Olsson et al^56^ found a greater cortisol awakening response in exhaustion disorder participants compared with healthy controls, and in another study by the same research group (of a partly overlapping sample) the response diminished in the exhaustion disorder group after treatment with *Rhodiola rosea* root.^90^ In studies by Sjörs et al^59,61^ no differences in the cortisol awakening response were reported between individuals with exhaustion disorder and healthy participants and no within-group changes were found during treatment.^59,61^ Similarly, Österberg et al^41^ found no difference in the cortisol awakening response between exhaustion disorder participants and healthy controls, but in that study lower evening cortisol levels in exhaustion disorder participants were reported. In a longitudinal study by Österberg et al,^42^ the cortisol awakening response in exhaustion disorder individuals was unchanged after 1.5 years compared with baseline. In studies by Jönsson et al^47^ and Lennartsson et al,^49^ no differences in cortisol response to versions of the Trier Social Stress Test were found when former exhaustion disorder participants were compared with a high-stress and a low-stress group or when exhaustion disorder participants were compared with a healthy control group.

Three studies by Lennartsson et al^48,50,51^ investigated the adrenal hormone DHEA-s, a precursor to androgens and oestrogens, in exhaustion disorder samples. In one study, lower DHEA-s response to the Trier Social Stress Test was found relative to healthy controls on one of the outcome measures used.^48^ When examining non-provoked levels of DHEA-s cross-sectionally between exhaustion disorder participants and controls, no main group differences were found, but levels of DHEA-s were lower in exhaustion disorder participants compared with controls in the youngest age group (25–34 years).^50^ In a longitudinal follow-up of the same sample, levels of DHEA-s were found to increase over time in half of the participants and decrease in the other half. Lower levels of self-rated symptoms of exhaustion were observed in the increase group.^51^

##### Growth factors and related molecules in plasma

Jonsdottir et al^46^ found no difference in epidermal growth factor (EGF), vascular endothelial growth factor (VEGF) or the chemokine MCP-1 between females with exhaustion disorder and controls. In a study by Wallensten et al,^65^ higher levels of VEGF and EGF were found in individuals with exhaustion disorder compared with healthy controls, but no difference in MCP-1. In contrast to these findings, Sjörs et al^62^ observed lower levels of VEGF and EGF in exhaustion disorder participants compared with healthy controls. Hadrevi et al^45^ analysed metabolomic patterns in plasma, reporting a difference in individuals with exhaustion disorder compared with healthy controls. Wallensten et al^66^ found higher levels of extracellular vesicles expressing the astrocytic marker glial fibrillary acidic protein (GFAP) in exhaustion disorder participants compared with individuals with depression and a previously collected sample of healthy controls, as measured using flow cytometry in plasma. In another analysis of the same sample, an elevation in exhaustion disorder individuals compared with controls was found for the VEGF_126_ isoform and total VEGF, but not for VEGF_165_.^67^ Total VEGF and VEGF_126_ were positively correlated with GFAP and aquaporin-4 (AQP4)-expressing extracellular vesicles.

##### Neuroimaging studies

Two studies investigated associations between exhaustion disorder status or symptom severity and brain volumetric measures assessed by magnetic resonance imaging (MRI). Savic et al^57^ found lower cortical thickness in areas of the right prefrontal cortex and left superior temporal gyrus in individuals with exhaustion disorder relative to controls. Among subcortical regions, smaller volumes of the caudate nucleus bilaterally and larger volumes of the right amygdala were found in exhaustion disorder participants. In a subgroup of individuals assessed after an average of 9 months of stress rehabilitation, individuals with exhaustion disorder showed an increase in thickness in the right prefrontal cortex compared with controls. Both groups showed a decrease in caudate volumes over time, but the decrease was significantly less pronounced in exhaustion disorder compared with controls.^57^ Malmberg Gavelin et al^55^ found smaller caudate volumes in exhaustion disorder participants who scored high on mental fatigue compared with those who scored low to moderate on mental fatigue. In another study by Savic et al,^58^ magnetic resonance spectroscopy was used to investigate different metabolite concentrations, and increased levels of glutamate were found in medial prefrontal cortex/anterior cingulate cortex in exhaustion disorder participants relative to controls.

Two studies investigated functional brain activity correlates of exhaustion disorder. Malmberg Gavelin et al^54^ used task-based functional magnetic resonance imaging (fMRI) in exhaustion disorder individuals and found that self-rated symptoms of exhaustion were positively associated with activation in several brain regions during the 2-back condition of an *n*-back task but not in the 3-back condition. In an exploratory longitudinal subgroup analysis of 10 participants before and after cognitive training added to stress rehabilitation, larger increases in activation were shown compared with participants who had not received such training.^54^ Skau et al^63^ examined exhaustion disorder individuals and healthy controls using functional near-infrared spectroscopy (fNIRS) during performance on six different cognitive tests. In a Stroop task, participants with exhaustion disorder showed attenuated blood flow compared with controls in areas of the left ventrolateral prefrontal cortex in the incongruent condition (i.e. when the target colour of words was incongruent with the written words) compared with the congruent condition.

##### Other biological measures

In a study by Sjörs et al,^60^ allostatic load, defined as a composite of 13 different biomarkers, was found not to differ in individuals with exhaustion disorder compared with healthy controls. Lindegård et al^53^ found an increase in an indirect measure of maximal oxygen uptake in a female sample with exhaustion disorder, which was non-linearly associated with reductions in self-rated symptoms of exhaustion over several time points across 18 months. In a cross-sectional study by Olsson et al,^56^ females with exhaustion disorder showed lower heart rate variability, higher temperature, lower peripheral O_2_ saturation and lower end-tidal CO_2_ levels compared with healthy female controls. Lennartsson et al^52^ also found lower heart rate variability in exhaustion disorder participants compared with healthy controls and compared with individuals with non-clinical burnout. Jönsson et al,^47^ however, found no difference in heart rate variability between individuals with and without exhaustion disorder at baseline or after the Trier Social Stress Test. In an uncontrolled study by Sonntag-Öström et al,^64^ exposure to forest environments was associated with lower heart rate compared with exposure to a city environment in individuals with exhaustion disorder. Ekstedt et al^44^ found that recovery after rehabilitation was associated with improvement on several sleep parameters as measured with polysomnography in individuals with exhaustion disorder.

##### Summary and critical appraisal

Studies of HPA-axis measures, of which some were well powered, suggest no differences between individuals with and without exhaustion disorder despite the proposed key role of a dysregulated stress response in the disorder. Similarly, the limited number of studies investigating growth factors such as EGF and VEGF do not collectively indicate differences between individuals with and without exhaustion disorder. Regarding other outcomes, attempts at direct replication of findings are few and many studies had an exploratory design. In the two published structural MRI studies, lower volumes of caudate nuclei were found in individuals with exhaustion disorder compared with healthy controls and in exhaustion disorder participants who scored high versus low on mental fatigue. Several studies suffered from methodological limitations, including limited statistical power and lack of adequately matched control samples. In some neuroimaging studies there was a lack of customary correction for multiple comparisons. Especially in the four studies without a control group, no conclusions can be drawn regarding biological correlates that may be specific to exhaustion disorder.

#### Symptom measurement scales

Four studies investigated psychometric properties of symptom measurement scales in exhaustion disorder samples. In a cross-sectional study using a matched healthy control group, Beser et al^69^ found the Karolinska Exhaustion Disorder Scale (KEDS) to be a unidimensional scale and that a cut-off score of 19 discriminated between individuals with exhaustion disorder and healthy controls with a sensitivity and specificity above 95%. Lundgren-Nilsson et al^70^ evaluated the construct validity of the 22-item version of the Shirom–Melamed Burnout Questionnaire (SMBQ-22) using both confirmatory factor analysis and Rasch analysis and identified problems with the scale's dimensionality. A revised version (18 items after removal of the ‘tension scale’) was found to provide better model fit, and a cut-off of 4.4 separated clinical from non-clinical cases with a sensitivity and specificity of over 83%.^70^ In another publication by the same research group,^71^ the construct validity of the 22-item Psychological General Well-Being Index (PGWI) was analysed in a sample of participants with exhaustion disorder at baseline and 3 months. The total score on the PGWI was deemed to summarise the well-being profile of most participants, with no significant difference in well-being by either age or gender. A significant improvement in well-being was observed at the 3-month assessment. Axelsson et al^68^ conducted a psychometric evaluation of the 12-item self-report version of the World Health Organization Disability Assessment Schedule 2.0 (WHODAS 2.0) when administered online to patients with health anxiety and stress disorders (exhaustion disorder and adjustment disorder). Individuals with exhaustion disorder were found to score significantly higher on the WHODAS 2.0 (indicating higher disability) compared with those with an adjustment disorder diagnosis.

##### Summary and critical appraisal

The KEDS and the SMBQ likelydiscriminate between those who have received an exhaustion disorder diagnosis and healthy individuals. The WHODAS 2.0 is indicated to discriminate between individuals with adjustment disorder and exhaustion disorder. There is a need for external validation and more studies investigating whether these self-rating scales can differentiate exhaustion disorder from other psychiatric or somatic conditions.

#### Interventions

Twenty-nine studies investigated interventions for exhaustion disorder. Of these, eight were original RCTs^76,79,83,87,90,92,96,98^ and seven were publications on subgroups or secondary analyses based on four of the RCTs.^74,77,84–86,93,94^ One prospective longitudinal study using matched controls was conducted,^80^ presenting a separate publication on long-term follow-up of participants,^81^ and a pre-test–post-test assessment of another intervention was conducted using a healthy control group as comparison.^91^ Of remaining studies, four were uncontrolled cohort studies,^78,82,88,100^ five were qualitative interview studies^72,73,75,97,99^ and two used mixed methods.^89,95^ The longest follow-up period of an intervention was 2.5 years from baseline. Supplementary Tables S3a and S3b give details about the primary publications of quantitative studies investigating interventions for exhaustion disorder.

##### Cognitive and behavioural interventions

In an RCT by Salomonsson et al,^92,93^ participants with common mental disorders were randomised to cognitive behavioural therapy (CBT), a work-directed psychological intervention (return-to-work intervention, RTW-I) or a combination of CBT and RTW-I. Individuals with exhaustion disorder (59% of the total sample) who were randomised to CBT showed reduced clinician-rated symptom severity with a moderate between-group effect size pre- to post-intervention compared with those randomised to RTW-I. The combined treatment was neither superior nor inferior to CBT or RTW-I regarding symptom reduction. At a 1-year follow-up no differences between treatments remained. When the same CBT intervention was tested as an internet-based treatment (ICBT) compared with a waiting list control group in an RCT by Lindsäter et al,^83^ large between-group effect sizes were found on the primary outcome of perceived stress as well as on secondary outcomes of exhaustion symptoms and insomnia severity. Mediation analyses in the two studies indicated that reduced insomnia severity mediated the effect of CBT compared with control conditions on symptoms of exhaustion^85,94^ and level of perceived stress.^85^ Finnes et al^76^ conducted an RCT in which the effect of acceptance and commitment therapy (ACT), a workplace dialogue intervention (WDI) and a combination of ACT and WDI were compared with treatment as usual (TAU) for individuals with common mental disorders (57% of whom were diagnosed with exhaustion disorder). Secondary symptoms of depression, anxiety and exhaustion improved with small to moderate effect sizes after ACT and the combined intervention relative to TAU pre- to post-intervention. There were no differences in symptoms between intervention groups and TAU at follow-up after 9 months. Two health-economic studies have been conducted, indicating that ICBT^84^ and ACT^77^ are cost-effective treatments relative to their respective controls from a societal and healthcare perspective.

##### Work-place interventions/return-to-work

Karlson et al^80^ found that, after a brief ‘convergence dialogue meeting’ intervention (CDM; discussion between the person and their workplace supervisor to improve the job–person match), more individuals returned to work (defined as 25% working time or more) over the study period compared with matched controls. There were, however, no differences between groups regarding full work resumption at the 1.5-year follow-up. An additional 1 year later, there was still no difference between groups in full work resumption or in relapses into sick leave.^81^ When a similar work-dialogue intervention was studied in the RCT by Finnes et al,^76^ no differences were found in net days of sickness absence between the intervention groups relative to TAU pre- to post-intervention. During the 9-month follow-up period, however, the combined treatment (ACT + WDI) generated more sickness absence compared with TAU. In the RCT by Salomonsson et al,^92^ the RTW-I generated no additional effect on net days of sickness absence compared with CBT or CBT in combination with RTW-I for sick-listed individuals with exhaustion disorder.^93^ An interview study of participants after completing a dialogue-based workplace intervention nevertheless reported that support in the return-to-work process was perceived by participants as important for moving towards feelings of empowerment and confidence.^99^

##### Multimodal rehabilitation

One uncontrolled cohort study investigated the effect of a 24-week standardised multimodal rehabilitation programme (MMR) for individuals with exhaustion disorder.^100^ The programme consisted of a combination of CBT group therapy, applied relaxation in group, individual psychotherapy, physiotherapy, lectures and medical treatment. A significant large within-group improvement on the main outcome of exhaustion symptoms was found post-treatment and was maintained to the 1-year follow-up.

##### Physical exercise

In a study by Lindegård et al,^82^ physically inactive participants with exhaustion disorder who complied with general instructions for physical exercise given within MMR showed reduced symptoms of exhaustion with a small effect size 18 months after treatment start compared with participants who did not comply. Gerber et al^78^ found no differences in self-reported physical activity after MMR when comparing a group who received a coached exercise programme with those who only received instructions to exercise. Both groups, however, reported increased exercise compared with baseline measurement. In an RCT by Eskilsson et al,^74^ group-based cycle training (as an add-on to MMR) was associated with a significant effect on a test of episodic memory post-intervention when compared with MMR only, but no effects on other cognitive domains or self-rated symptoms were found. At a 1-year follow-up, there were no differences between groups regarding cognitive functioning or self-rated symptoms.^87^

##### Cognitive training

In an RCT by Malmberg Gavelin et al,^75,86,87^ participants who received a cognitive training programme as a complement to MMR showed a small, significant improvement on the trained memory task as well as small improvements on three of nine transfer tests (executive functioning and episodic memory) post-intervention, relative to participants who received only MMR.^86^ At a 1-year follow-up, participants in the cognitive training group showed sustained improvement on the trained memory task, but also a small transfer effect in the form of improved global cognitive score relative to controls.^87^ No differences between groups were found on the separate cognitive tests that were reported in the first publication, and no between-group differences were found on self-rated symptom domains at follow-up.^87^

##### Nature/gardening/horticulture

In an uncontrolled pilot study by Sonntag-Öström et al,^95^ more positive ratings of mental state were reported after 11 weeks of visiting different forest environments. A subsequent RCT comparing a forest rehabilitation group and a waiting list control group showed no differences between groups on mental health outcomes or sick leave.^96^ In interviews participants described the intervention as ‘insufficient as rehabilitation’.^97^ In an uncontrolled study by Nordh et al,^89^ an intervention teaching recreational activities in forest environments was found to reduce stress but had no significant effect on symptoms of exhaustion or functioning and was associated with a significant reduction in quality of life at the end of the intervention. Two interview studies by Adevi and colleagues indicated that a 12-week multimodal therapy focused on nature and traditional gardening and handicraft activities was perceived by rehabilitation personnel to be helpful for recovery of exhaustion disorder participants,^72^ whereas exhaustion disorder participants who were interviewed reported that the recovery process needs to be initiated by more traditional forms of therapy.^73^ Millet et al^88^ found small improvements in mental health outcomes in an uncontrolled pilot study investigating the integration of horticulture into vocational rehabilitation.

##### Alternative and complementary treatments

A double-blind RCT by Olsson et al^90^ found *Rhodiola rosea* extract to reduce the primary outcome of exhaustion symptoms compared with placebo controls at follow-up after 28 days. Stenlund et al^98^ conducted another RCT and found no differences in primary or secondary health outcomes between participants who received qigong as a complement to care as usual and those who only received care as usual. In a third RCT, by Grensman et al,^79^ no differences between group treatments consisting of traditional yoga, mindfulness-based CBT or CBT were found on the main outcome of health-related quality of life. African dance as an intervention for exhaustion disorder was not associated with improvements on the main outcome of hyperventilation compared with no intervention in a non-randomised trial conducted by Ristiniemi et al,^91^ but a significant time × group interaction effect was found indicating positive effects on symptoms of exhaustion and depression.

##### Summary and critical appraisal

A multitude of interventions have been investigated for exhaustion disorder, but the evidence for any one type of intervention is limited. Obtained results suggest that sick leave decreases over time independent of intervention. Three RCTs indicate symptom reduction in exhaustion disorder participants after cognitive behavioural interventions (including ACT) relative to control conditions post-intervention, but no between-group differences remain at follow-ups. There is inconclusive evidence that cognitive training may have transfer effects on cognitive functioning in individuals with exhaustion disorder. Physical exercise in the treatment of individuals with exhaustion disorder has been insufficiently studied, as have various alternative and complementary treatments. Owing to methodological limitations (e.g. no control for confounding factors in non-randomised trials, generally small samples, unspecified primary outcomes, and significant attrition in some studies), no firm conclusions can be drawn about the effectiveness of interventions for individuals with exhaustion disorder. Notably, even though MMR is an intervention that is commonly used in clinical settings^23,87,99^ and is recommended by the Swedish National Board of Health and Welfare, only one study explicitly examined MMR, using a non-controlled design. No studies were found that examined the effect of seemingly common pharmacological treatments for exhaustion disorder, such as antidepressant medication.^26,33^

## Discussion

This is the first scoping review to systematically investigate the evidence base and knowledge gaps regarding the stress-related diagnosis exhaustion disorder in the Swedish version of the ICD-10. Even though chronic stress and fatigue constitute global challenges,^4^ exhaustion disorder has not been accepted into international versions of the ICD. The current review found 89 studies that explicitly studied exhaustion disorder, covering a broad range of research areas related to the lived experience of exhaustion disorder, symptom presentation and course, cognitive functioning, biological measures, self-rated symptom scales and treatment. The yearly rate of exhaustion disorder publications has increased substantially since the introduction of the diagnosis, yet the total number of studies and the number of studies within each thematic category remain limited. This review sheds light on the symptomatic heterogeneity of individuals diagnosed with exhaustion disorder, and our syntheses of findings suggest that few firm and generalisable conclusions can be drawn about the diagnosis.

### State of the knowledge base

This review is limited by the fact that the quality of the evidence was not systematically assessed using, for example, the GRADE rating criteria for each individual study. Nevertheless, several important methodological concerns were observed across included studies. First, samples were often small, and few studies specified primary outcomes and presented power calculations, which together make it difficult to identify reliable results. Second, healthy control groups used as comparisons commonly constituted convenience samples rather than population-representative samples, which limits validity and generalisability of findings. Third, the dominant use of cross-sectional and uncontrolled designs limits inferences about causality, and lack of comparisons with other diagnostic entities means that no conclusions can be drawn regarding the specificity of the exhaustion disorder diagnosis. Fourth, intervention studies, which are of particular importance to guide clinical practice, included only eight RCTs investigating the effect of different treatment strategies. A lack of adherence to the intention-to-treat principle and failure to use adequate methods to account for missing data were noted in some studies. Last, only a small minority of studies reported preregistered study protocols to establish hypotheses, primary outcome measures and a prespecified plan for data analysis. This introduces potential publication bias as well as measurement and reporting bias and hampers the reproducibility of findings. These methodological considerations, together with the limited number of studies, indicate that the exhaustion disorder research field is still in its infancy.

Thirty-nine studies, primarily conducted in Sweden, were excluded from the synthesis because of unclear references to exhaustion disorder diagnostic criteria or because exhaustion disorder was self-assessed (supplementary Table S2). Examples of terminology used to define samples in these studies were ‘chronic occupational stress’,^101^ ‘severe occupational burnout’^102^ and ‘diagnosis of burnout’.^103^ Although it is possible that these samples overlap with exhaustion disorder samples, the different definitions presented in the excluded articles obstruct reliable synthesis of results. This highlights a recurring problem of diagnostic clarity in the field of chronic stress.^104^ Moreover, using cut-offs on self-rating scales to define an exhaustion disorder sample is problematic because no studies to date have investigated whether exhaustion disorder-specific symptom scales reliably distinguish between individuals with exhaustion disorder and those with other psychiatric and somatic conditions. For example, a recent Danish study found no significant differences on the self-rated Karolinska Exhaustion Disorder Scale (KEDS) between individuals with stress-related disorders and individuals with major depressive disorders,^105^ and the association between the exhaustion dimension and depression in a meta-analysis of burnout was found to be problematically strong from a discriminant validity standpoint.^106^

One obvious explanation for the limited number of studies of exhaustion disorder is that it is not included in international diagnostic systems. As a contrasting example, the stress-related diagnosis of adjustment disorder (ICD-10 diagnostic code F43.2) has been the topic of international debate for many years,^107–109^ which has contributed to propelling research forwards. Since revised diagnostic criteria for adjustment disorder were proposed for inclusion in ICD-11, more than 800 international publications have covered areas such as symptom structure,^110,111^ natural course^112,113^ and specificity of the diagnostic construct,^114–116^ as well as new assessment methods and measurement scales.^117,118^ This type of joint international research effort is not possible when investigating a uniquely Swedish diagnosis. Moving towards international cooperation is of major importance to make rapid progress in understanding the suffering and disability seen in individuals diagnosed with exhaustion disorder and other stress- and fatigue related conditions.

### Knowledge gaps

To date, several questions regarding the most basic assumptions of exhaustion disorder remain unanswered, such as the proposed aetiology (that symptoms develop in response to identifiable stressors) and the validity and reliability of exhaustion disorder criteria (e.g. fatigue, impaired cognition, emotional instability or irritability, insomnia or hypersomnia, and physical symptoms), that likely overlap a range of other psychiatric and somatic domains. Results from the current review indicate that many individuals diagnosed with exhaustion disorder experience clinically significant psychiatric and somatic symptoms and decreased quality of life compared with healthy controls, but how these symptoms compare with clinical expressions of other disorders remains largely uninvestigated. Also, even though many individuals diagnosed with exhaustion disorder self-report cognitive impairments, valid psychometric tests across studies detect mainly minor and inconsistent impairments. The same pattern of discrepancy is also manifest in studies investigating the physiological stress response. Regarding biological measures, no clear direction of findings across a range of outcomes has been observed. The prevalence of exhaustion disorder is largely unknown, as is the natural course of the condition. The limited understanding of the exhaustion disorder diagnosis precludes systematic investigations into, for example, the effect of specific interventions, and constrains development of evidence-based treatment guidelines.

### Implications for research and practice

The limited evidence for the exhaustion disorder diagnosis has implications for precision in prognosis and treatment, likely affecting afflicted individuals negatively. The present review calls for a change in priorities when it comes to exhaustion disorder research, moving towards increased national and international collaboration with the aim of investigating the validity and reliability of exhaustion disorder as a unique diagnostic construct that might encompass fatigue-dominated states seemingly triggered by exposure to chronic stressors. This involves empirical investigation into whether other topographically similar constructs such as burnout^6^ and fatigue,^9^ as well as other stress disorders (e.g. adjustment disorder), subtypes of depression, and anxiety disorders, have common psychological and biological underpinnings. Differential diagnostics is a key issue that needs to be better addressed in future studies to strengthen the knowledge base regarding exhaustion disorder as a unique diagnostic construct. Importantly, preregistration of trials and data-sharing should be prioritised to improve reproducibility, decrease publication bias, facilitate comparisons across studies and enable analysis of individual patient-level data. An international effort to establish a core outcome set (COS)^119^ to be used across clinical trials of individuals suffering from mental and physical exhaustion would facilitate the comparison and combination of results in treatment studies.

## Data Availability

All data underlying the results are available as part of the article and in the online supplement, and no additional source data are required. Extended data are presented in the online supplement.

## Supplementary material

For supplementary material accompanying this paper visit http://doi.org/10.1192/bjo.2022.559. click here to view supplementary material

## Appendix

Diagnostic criteria for exhaustion disorder (F43.8A) published by the Swedish National Board of Health and Welfare (ICD-10-SE). Criteria A–F must all be met to set the diagnosis.

- Physical and mental symptoms of exhaustion for at least 2 weeks. The symptoms have developed in response to one or more identifiable stressors, which have been present for at least 6 months.
- Markedly reduced mental energy, manifested by reduced initiative, lack of endurance or increase in time needed for recovery after mental efforts.
- At least four of the following symptoms have been present most of the day, nearly every day, during the same 2-week period:
  1. persistent complaints of impaired memory and concentration
  2. markedly reduced capacity to tolerate demands or to perform under time pressure
  3. emotional instability or irritability
  4. insomnia or hypersomnia
  5. persistent complaints of physical fatigue and lack of endurance
  6. physical symptoms such as muscular pain, chest pain, palpitations, gastrointestinal problems, vertigo or increased sensitivity to sounds.
- The symptoms cause clinically significant distress or impairment in social, occupational or other important areas of functioning.
- The symptoms are not due to the direct physiological effects of a substance (e.g. misuse of a drug or medication) or a general medical condition (e.g. hypothyroidism, diabetes, infectious disease).
- The stress-related disorder does not meet criteria for major depressive disorder, dysthymic disorder or generalised anxiety disorder. [If, for or example, criteria for depression are met, exhaustion disorder is to be used as a complementary diagnosis.]
