# Exhaustion disorder: scoping review of research on a recently introduced stress-related diagnosis – ERRATUM

**DOI:** 10.1192/bjo.2022.581

**Published:** 2022-09-29

**Authors:** Elin Lindsäter, Frank Svärdman, John Wallert, Ekaterina Ivanova, Anna Söderholm, Robin Fondberg, Gustav Nilsonne, Simon Cervenka, Mats Lekander, Christian Rück

**Keywords:** Review, psychological stress, fatigue, burnout, nosology

The corresponding author's email address was misspelled in the above paper. The correct email is: elin.lindsater@ki.se

The Publisher apologises for the error.
