# Supplementary material for: Exhaustion disorder: scoping review of research on a recently introduced stress-related diagnosis
Source: BJPsych Open. 2022 Aug 24;8(5):e159. doi: 10.1192/bjo.2022.559 (PMC9438479; doi:10.1192/bjo.2022.559)
Supplement: Supplementary file 1 [file S2056472422005592sup001.pdf]

SUPPLEMENTARY MATERIAL

Exhaustion Disorder: A Scoping Review of Research on a Recently Introduced Stress-Related Diagnosis

Elin Lindsäter[1,2*], Frank Svärdman[1], John Wallert[1], Ekaterina Ivanova[1], Anna Söderholm[3], Robin Fondberg[1], Gustav Nilsonne[2,4], Simon Cervenka[1,5], Mats Lekander[2,4,6], Christian Rück[1]

[1] Center for Psychiatry Research, Department of Clinical Neuroscience, Karolinska Institutet, & Stockholm Health Care Services, Region Stockholm, Sweden
[2] Division of Psychology, Department of Clinical Neuroscience, Karolinska Institutet, Stockholm, Sweden
[3] Department of Psychology, Umeå Universitet, Umeå, Sweden.
[4] Stress Research Institute, Department of Psychology, Stockholm University, Stockholm, Sweden
[5] Department of Medical Sciences, Psychiatry, Uppsala University, Uppsala, Sweden
[6] Osher Center for Integrative Medicine, Department of Clinical Neuroscience, Karolinska Institutet, Stockholm, Sweden

*Corresponding author: Elin Lindsäter, Department of Clinical Neuroscience, Karolinska Institutet. Gustavsberg Primary Care Clinic, Odelbergs väg 19, SE-134 40 Gustavsberg, Sweden
E-mail: elin.lindsater@ki.se

**Content**	Pages
**Table S1**. Full search strategy	*2*
**Table S2.** Overview of excluded but “complementary” studies	*3-6*
**Tables S3a and S3b**. Data-charting of primary publications of quantitative studies investigating exhaustion disorder treatment	*7-10*
**Table S4**. Overview of included empirical studies of individuals diagnosed with exhaustion disorder, ED	*11-17*
**Table S5**. Overview of dissertations	*18-20*
**References**	*21-27*

Table S1. Full search strategy

Search Strategy†	Medline	Psycinfo	Web of Science
Interface	Ovid	Ovid	Clarivate Analytics
No of hits	3691	2179	1804
Field labels	_ exp/ = exploded MeSH term<br>_ / = non exploded MeSH term<br>_ * = truncation of word for alternate endings<br>_ mp. = title, abstract, original title, name of substance word, subject heading word, protocol supplementary concept word, rare disease supplementary concept word, unique identifier<br>_ in. = institution	_ exp/ = exploded controlled term<br>_ / = non exploded controlled term<br>_ * = truncation of word for alternate endings<br>_ mp = title, abstract, heading word, table of contents, key concepts, original title, tests measures<br>_ in. = institution	_ TS = topic<br>_ AD = address<br>_ * = truncation of word for alternate endings
1.	exp Stress, Psychological/	exp Stress/	TS=burnout
2.	exp Adjustment Disorders/	exp Adjustment Disorders/	TS=exhaustion
3.	burnout.mp.	burnout.mp.	TS="psychologic* stress*"
4.	exhaustion.mp.	exhaustion.mp.	TS="occupational stress*"
5.	1 or 2 or 3 or 4	1 or 2 or 3 or 4	TS="adjustment disorder*"
6.	sweden.mp. or exp Sweden/	sweden.mp. or exp Sweden/	5 OR 4 OR 3 OR 2 OR 1
7.	(sweden or lund or linkoping or stockholm or gothenburg or umea or uppsala or karlstad or orebro or ostersund or vaxjo).in.	(sweden or lund or linkoping or stockholm or gothenburg or umea or uppsala or karlstad or orebro or ostersund or vaxjo).in.	AD=(sweden OR lund OR linkoping OR stockholm OR gothenburg OR<br>umea OR uppsala OR karlstad OR orebro OR ostersund OR vaxjo)
8.	6 or 7	6 or 7	7 AND 6
9.	5 and 8	5 and 8	TS=("exhaustion disorder*")
10.	exhaustion disorder*.mp.	exhaustion disorder*.mp.	TS=("exhaustion syndrome*")
11.	exhaustion syndrome*.mp.	exhaustion syndrome*.mp.	TS=("exhaustion depression*")
12.	exhaustion depression*.mp.	exhaustion depression*.mp.	11 OR 10 OR 9
13.	10 or 11 or 12	10 or 11 or 12	12 OR 8
14.	9 or 13	9 or 13	-

†All searches were initially conducted on June 17th 2020 with the aid of librarian Carl Gornitzki, Universitetsbiblioteket, Karolinska Institutet

Table S2. Overview of excluded but "complementary" studies (*N* = 39) with unclear reference to, or self-rated, exhaustion disorder (ED).

First author	Year	Design	Aim/question
Lived experience of ED, n = 2			
Gustafsson[1]	2008	Qualitative	To illuminate the meanings of becoming and being burned out as narrated by healthcare personnel on sick leave because of symptoms of burnout.
Håkansson[2]	2010	Qualitative	To re-analyze the data for its congruence with the Matuska and Christiansen life balance model using a matrix system, and to test the validity of the model.
Symptoms, course and context, n = 10			
Asplund[3]	2021	Cross-sectional	To assess the prevalence of self-rated exhaustion disorder (s-ED), describe plausible between-group differences in self-reported health-related factors among employees with or without s-ED, and identify health-related factors associated with s-ED.
Broddardottír[4]	2021	Cross-sectional	To identify ED patients whose fatigue meets criteria for "persistent physical symptoms" (PPS) and explore whether they differ from other ED patients in terms of psychological distress, non-fatigue PPSs and functional impairment, inspecting whether this alternative formulation of the fatigue problem might be more appropriate.
Gustafsson[5]	2009	Cross-sectional, case control	To describe patterns of personality traits among two groups of health-care personnel from the same workplaces, one group on sick leave due to medically assessed burnout, and one group with no indication of burnout, respectively
Gustafsson[6]	2010	Cross-sectional, case control	To elucidate perceptions of conscience, stress of conscience, moral sensitivity, social support and resilience among two groups of health care personnel from the same workplaces, one group on sick leave owing to medically assessed burnout and one group who showed no indications of burnout.
Håkansson[7]	2018	Longitudinal, cohort	To investigate whether perceived occupational imbalance predicts stress-related disorders and possible gender differences. To explore the mediating role of perceived stress in the association between occupational imbalance and stress-related disorders.
Höglund[8]	2020	Cross-sectional	To determine symptom severity of anxiety, depression, insomnia, burnout and somatization in combinations of different age groups and sex. To determine prevalence of caseness of these types of mental ill-health in both absolute and relative terms in the combinations of age groups and sex.
Norlund[9]	2011	Cohort, prospective	To investigate the impact of psychosocial working conditions and coping strategies at work on change in sick leave level for patients on long-term sick leave due to burnout.

Stenlund[10]	2007	Case control	To describe gender differences in patients with burnout and compare these patients with a general population with respect to physical, psychosocial and work variables.
Söderström[11]	2012	Prospective	To identify risk factors for subsequent clinical burnout.
Wiegner[12]	2015	Observational, Cohort	To examine to what extent a working age population seeking primary health care perceives stress, as well as symptoms of burnout/exhaustion, depression and anxiety.
Cognitive functioning, n = 1			
Sandström[13]	2005	Cross-sectional, case control	To conduct a thorough examination of the cognitive performance of patients with a diagnosis of chronic burnout
Biological measures, n = 10			
Blix[14]	2013	Cross-sectional, case control	To examine whether chronic work-related stress is associated with changes in brain structure.
Bäckström[15]	2012	Cross-sectional, case control	To compare GABA-A receptor sensitivity as indexed by maximal saccadic eye velocity between burnout and control subjects
Ekstedt[16]	2006	Repeated measures, case control	To investigate sleep with polysomnography and self-ratings and the diurnal pattern of sleepiness and fatigue in a group suffering from severe occupational burnout.
Golkar[17]	2014	Cross-sectional, case control	To investigate whether (1) subjects suffering from occupational stress have an impaired ability to modulate stressful emotions; and whether (2) these subjects show altered amygdala functional connectivity
Grossi[18]	2005	Repeated measures, case control	To compare salivary cortisol awakening response between individuals with low, moderate and high burnout scores
Heiden[19]	2005	Cross-sectional, case control	To characterize patients with stress-related illnesses by comparing autonomic activity, pressure-pain thresholds, and subjective assessments of health and behavior between patients with stress-related illnesses and control subjects.
Jovanovic[20]	2011	Repeated measures, case control	To investigate whether enduring daily stress causes widespread limbic dysfunctions, and specific changes of the 5-HT1A receptor.
Sandström[21]	2011	Cross-sectional, case control	To use a multivariate statistical approach to examine whether patients with work-related exhaustion and controls differed on an extensive set of biological, psychological and immunological variables.
Sandström[22]	2012	Cross-sectional, case control	To compare functional magnetic resonance imaging (fMRI) patterns and diurnal cortisol across three groups: (i) controls, (ii) acute un-medicated patients with unipolar major depression, and (iii) patients on long-term sick leave due to work stress.
Savic[23]	2015	Repeated measures, case control	To compare salivary cortisol, cortical thickness, cortical surface area and subcortical volumes between individuals with occupational stress compared to controls.

Symptom measurement scales, n = 4			
Glise[24]	2010	Longitudinal, cohort	To assess the construct and predictive validity of a new instrument for self-rating of stress-related Exhaustion Disorder (s-ED).
Persson[25]	2016	Cross-sectional	To benchmark the Lund University Checklist for Incipient Exhaustion (LUCIE) against the s-ED and the Karolinska Exhaustion Disorder Scale, but also against other ED-related concepts such as burnout.
Persson[26]	2017	Cross-sectional	To examine the relationships of two screening instruments recently developed for assessment of ED with some other well-known inventories intended to assess ED-related concepts and self-reports of job demands, job control, job support, private life stressors, and personality factors.
Saboonchi[27]	2013	Cross-sectional	To examine the psychometric properties of Karolinska Exhaustion Scale (KES) in its original and revised versions by examining the factorial structure and measures of convergent and discriminant validity.
Interventions, n = 12			
Cerwén[28]	2016	Qualitative	To increase understanding of the role of soundscapes in Nature Based Rehabilitation (NBR),
Fjellman-Wiklund[29]	2010	Qualitative	To explore patients' experiences in a burnout rehabilitation programme with two different rehabilitation groups.
Grahn[30]	2017	Longitudinal, cohort	To examine return to work a year after the start of participation in nature-based rehabilitation programs with different lengths (8, 12, and 24 weeks).
Grossi[31]	2009	Longitudinal, case control	To determine whether complementary therapy based on a group treatment program could improve the patients' health, physiological markers and work capacity better than the standard individual treatment program offered by the municipal company healthcare.
Heiden[32]	2007	RCT	To evaluate the effects of a cognitive behavioural training programme and a physical activity programme, compared with usual care, for patients with stress-related illnesses.
Nygren[33]	2019	Longitudinal, case control	To examine whether a combination of a multimodal rehabilitation, group-talks with collegues, and active monitoring (intervention carried out on an all-inclusive hotel in Gran Canaria) leads to improved return-to-work rates in sick-listed teachers with exhaustion disorder compared with treatment as usual.
Pálsdóttir[34]	2014	Mixed method	To describe and assess changes in participants' experiences of everyday occupations after nature-based vocational rehabilitation and to assess changes regarding symptoms of severe stress and the rate of return to work and possible association with experiencing the occupational value of everyday occupations.
Pálsdóttir[35]	2014	Qualitative, longitudinal	To explore and illustrate how participants with stress-related mental disorders participating in nature-based rehabilitation experience and describe their rehabilitation process in relation to the role of the natural environments.

Person-Asplund[36]	2018	RCT	To evaluate the efficacy of a guided internet-based stress management intervention among distressed managers compared with an attention control group with full access to treatment-as-usual.
Sahlin[37]	2015	Longitudinal, cohort	To explore the effects of nature-based rehabilitation in patients with exhaustion disorder or stress-related mental disorders.
Stenlund[38]	2009	RCT	To evaluate effects on psychological variables and sick leave rates by two different group rehabilitation programs for patients on long-term sick leave because of burnout.
Stenlund[39]	2012	RCT, secondary publication	To evaluate the long-term effects of two different rehabilitation programs for patients on long-term sick leave for burnout.

RCT, randomized controlled trial

Table S3a. In-depth data-charting of primary publications of quantitative studies included in the review that investigated interventions for exhaustion disorder.

First author, year	Pre-registration of trial	Design	N	Intervention (*n*)	Control (*n*)	Age (mean)	Women (%)	% ED
Eskilsson, 2017[40]	No	RCT†	88	Multimodal rehabilitation (MMR)+ aerobic training (AT) (47)	MMR (41)	42	88	100
Finnes, 2017[41]	Yes	RCT	352	Acceptance and commitment therapy (ACT) (90); Workplace dialogue intervention (WDI) (90); ACT+WDI (90)	Treatment as usual (89)	46	78	67
Gerber, 2015[42]	No	Cohort	169	MMR+coached exercise (36)	MMR (133)	43	79	100
Grensman, 2018[43]	Retrospective	RCT	94	Traditional yoga (TY) (26); Mindfulness-based CBT (MBCBT) (27)	CBT (27)	44	89	100
Karlson, 2010[44]	Yes	Prospective case-control	148	Convergence diaglogue meeting (CDM) (74)	Care as usual (74)	46	78	89
Lindegård, 2015[45]	No	Cohort	69	MMR (69)	N/A	43	65	100
Lindsäter, 2018[46]	Yes	RCT	100	Internet-based CBT (ICBT) (50)	Waitlist control (50)	46	85	47
Malmberg Gavelin, 2015[47]	No	RCT†	99	MMR+cognitive training (CT) (53)	MMR (46)	43§	85	100
Malmberg Gavelin, 2018[48]	Retrospective	RCT†	132	MMR+CT (44); MMR+AT (47)	MMR (41)	43§	84	100
Millet, 2009[49]	No	cohort	32	Nature/Gardening (32)	N/A	46 (median)	100	100
Nordh, 2009[50]	No	cohort	24‡	Forest rehabilitation (24)	N/A	45	57	48
Olsson, 2009[51]	No	RCT	60	Rhodiola rosea (30)	Placebo (30)	42§	90	100
Ristiniemi, 2014[52]	No	Pre-post, case-control	44	African dance (15)	Healthy controls (14); ED controls (15)	44§	82	100
Salomonsson, 2017[53]	Yes	RCT	211	CBT (64); CBT+Return to work intervention (RWT-I) (80)	RTW-I (67)	42	82	59
Sonntag-Öström, 2015[54]	No	RCT	99	Forest rehabilitation + MMR (51)	Waitlist + MMR (48)	45	86	100
Stenlund, 2009[55]	No	RCT	82	Qigong (41)	Basic care (41)	44	83	100
van de Leur, 2020[56]	Yes	Cohort	390	MMR (390)	N/A	44	88	100

RCT, randomized controlled trial; CBT, cognitive behavior therapy

[†]Same RCT reporting on different outcomes. Inconsistencies in reported number of participants in the three publications from the same RCT.
[‡]Only baseline data on 21 participants.
[§]Mean age only reported for subgroups. Mean age for total sample calculated by research team (mean across subgroups).

Table S3b. Table S3a continued, illustrating additional data-charting from primary publications of quantitative studies included in the review.

**First author, year**	**Length of treatment (weeks)**	**Post-assessment follow-up (months)**	**Primary outcome(s)**	**% attrition to post-assessment (Intervention)**	**% attrition to post-assessment (Control)**	**ITT** †	**Selected main finding**
Eskilsson, 2017[40]	12 MMR + 12 MMR+AT	no	Cognitive test battery	49	22	no	Episodic memory improved in MMR+AT vs control. No differences on self-reported mental and physical health outcomes.
Finnes, 2017[41]	12	3, 9	Net days on sick leave (registry), work ability index	ACT: 27; WDI:42; ACT+WDI: 28	27	yes	No difference in sickness absence (SA) post-assessment. At 9m follow-up, more SA in ACT+WDI compared to TAU. ACT and ACT+WDI improved symptoms more than TAU at post-assessment.
Gerber, 2015[42]	52	6, 12	Self-reported frequency, duration, and intensity of exercise	0	0	N/A	No differences between coached and general exercise advice. All participants reported increased exercise frequency.
Grensman, 2018[43]	20	no	Health-related quality of life (SWED-QUAL)	TY: 19; MBCT: 13	CBT: 13	no	All group treatments had equal positive effects on health-related quality of life.
Karlson, 2010[44]	unclear	weekly until week 80	Return-to-work; yes/no (registry)	N/A	N/A	N/A	More participants in the intervention-group had returned to work (89%) after 1,5 years compared to control group (73%). No difference between groups regarding full return to work.
Lindegård, 2015[45]	52	6, 12, 18	Self-reported frequency, duration, and intensity of exercise	0	N/A	N/A	Higher compliance with physical activity recommendation was associated with decreased levels of ED symptoms and depression.
Lindsäter, 2018[46]	12	6	Perceived Stress Scale-14	2	4	yes	ICBT vs waitlist made large and significant improvements on PSS-14 (d = 1.09).
Malmberg Gavelin, 2015[47‡]	12 MMR + 12 MMR+CT	no	Cognitive test battery	49	30	no	Significant small effects on three of nine transfer tests at post-intervention in MMR+CT vs MMR only.

Malmberg Gavelin, 2018[48‡]	12 MMR + 12 MMR+CT Or 12 MMR+AT	12	Global cognitive performance on cognitive test battery	CT:36; AT: 49	20	yes	Higher global cognitive score in the MMR+CT group vs MMR only. No between-group differences in other psychological assessments.
Millet, 2009[49]	21 to 29	no	Not stated	0	N/A	N/A	Reductions in stress and cortisol levels and improvements in sleep and energy post treatment.
Nordh, 2009[50‡]	10	no	Not stated	N/A	N/A	N/A	Decrease in stress (SCI) and quality of life and an increase in anxiety and depressed mood post-treatment.
Olsson, 2009[51]	4	no	Pines' burnout scale	3	0	no	Small between-group effects on Pines in favor of the treatment.
Ristiniemi, 2014[52]	4	no	Nijmegen Symptom Questionnaire	0	0	N/A	Higher levels of hyperventilation in ED participants vs healthy controls pre-intervention. No between-group effects on hyperventilation at post-intervention assessment.
Salomonsson, 2017[53]	8-20 CBT; up to 25 CBT+RTW-I; up to 10 RTW-I	6, 12	Net days on sick leave (registry); Clinician Severity Rating	CBT: 8; CBT+RTW-I: 6	RTW-I: 12	yes	No differences in sick leave. CBT reduced clinican rated symptom severity vs RTW-I at post-intervention. No additional benefit of CBT+RTW-I on symptom reduction.
Sonntag-Öström, 2015[54]	12 forest rehabilitation + 24 MMR	9	Not stated	22	4	no	No significant between-group differences for any of the variables.
Stenlund, 2009[55]	12	no	Shirom Melamed Burnout Questionnaire-22	20	15	yes	No significant between-group differences for any of the variables.
van de Leur, 2020[56]	24	12	Karolinska Exhaustion Disorder Scale; self-rated sick leave	3	N/A	N/A	Large symptom reduction post treatment and at follow-up, increased self-reported working time and reduced sick-leave compensation.

[†]ITT, Intention to treat analysis

[‡]Information about attrition cannot be estimated based on data in the publication.

Table S4. Overview of included empirical studies of individuals diagnosed with exhaustion disorder, ED (N = 89)

First author	Year	Design	Sample size	Women %	Aim of the study
Lived experience of Exhaustion Disorder (ED), n = 9					
Alsén[57]	2020	Qualitative	12	58	Explore ED participants' experience of ED in the early stages of sick leave.
Arman[58]	2011	Qualitative	18	67	Get a deeper and existential understanding of burnout, by looking at patterns of health, suffering and expressions of understanding of life in a longitudinal perspective.
Engebretsen[59]	2018	Qualitative	8	75	Assess how the values that go with the biomedical framework affect medical inquiry and the attitudes of the medical profession related to how burnout is understood and treated.
Engebretsen[60]	2019	Qualitative	8	75	To describe the experience of suffering from burnout while waiting to be recognized as ill, as the diagnosis "Exhaustion disorder" is not recognized in Norway.
Engebretsen[61]	2020	Qualitative	8	75	Explore how ED participants on long-term sick leave deal with the process of coming to terms with their present body in the rehabilitation process.
Ericson-Lidman[62]	2007	Qualitative	15	100	Describe co-workers' perceptions of signs preceding ED in workmates.
Hörberg[63]	2020[†]	Qualitative	12	100	Describe how women with stress-related illness experience well-being in everyday life.
Jingrot[64]	2008	Qualitative	11	73	Explore the lived experiences of the process leading to ED.
Norlund[65]	2013	Qualitative	12	83	Explore experiences and thoughts in the process of returning to work in employed individuals with ED.
Symptoms, course, and context, n = 13					
Adamsson[66]	2018	Retrospective medical chart	115	77	Investigate the frequency of different stress-related complaints present 2 years prior the confirmation of ED diagnosis.
Beno[67]	2021	Cross-sectional	217	74	To explore whether participants with ED had made any changes in their work situation from the period of treatment and up to 7 years later, as reported at the follow-up.
Glise[68]	2012	Longitudinal, cohort	228	68	Explore the course of illness (primarily symptoms of burnout) for 18 months among individuals diagnosed and treated for ED and if course of illness was related to sex and age.
Glise[69]	2014	Longitudinal, cohort	228	68	Explore the prevalence of somatic symptoms in individuals with ED and follow the course of symptoms for 18 months while participating in a multimodal rehabilitation program.
Glise[70]	2020	Longitudinal, cohort	217	74	To explore perceived recovery, and residual symptoms including fatigue, depression, and anxiety among previous ED patients 7 years after seeking care.
Grensman[71]	2016	Cross-sectional, case control	ED 92; Control 88	84	Explore the health-related quality of life (HRQoL), the cause of being ill, and the pharmacological treatment in individuals on sick leave because of ED.

Grossi[72]	2015	Cross-sectional	420	77	Investigate differences in socio-demographic variables, use of medications, quality of sleep and symptoms of anxiety, depression, and fatigue in a sample of Swedish men and women referred to care for ED.
Grossi[73]	2021	Cross-sectional	808	84	Assess the prevalence of self-rated hazardous drinking in individuals with ED, and to investigate differences in sociodemographic variables, psychological symptoms, health-related quality of life, and sleep variables between individuals with different drinking patterns.
Gulin[74]	2021	Cross-sectional	147	78	To investigate whether recovery from ED is associated with obsessive-compulsive personality disorder.
Hasselberg[75]	2014	Mixed method	Part 1: 20<br>Part 2:100	50	Explore which stressors are reported as important for the onset of illness by individuals seeking medical care for ED, the prevalence of these stressors, and potential gender differences.
Maroti[76]	2017	Cross-sectional, case control	ED 31;<br>CFS 38;<br>Control 30	80	Investigate if there are differences between ED and Chronic fatigue syndrome (CFS) in reaction to self-reported alexithymia and observer-rated emotional awareness.
Maroti[77]	2018	Cross-sectional, case control	ED 31;<br>CFS 38;<br>Control 30	80	Compare quality of life between individuals with ED, CFS, and healthy controls (HC) by using the SF-36 and HADS.
Skoglund[78]	2018[†]	Prospective, medical chart	192	79	To verify if individuals on antidepressant therapy and on long-term sick leave for mild and moderate depression, anxiety, and stress-related mental disorders have a longer sick leave than individuals treated with psychological and other therapies.
Cognitive functioning, n = 10					
Bartfai[79]	2021	Cross-sectional	39	90	To explore the diagnostic potential of the MapCog Spectra and validate the results through simultaneously obtained data on clinical neuropsychological tests.
Ellbin[80]	2018	Cross-sectional, case control	ED 93;<br>Control 111	69	Determine whether a brief test battery such as CAB (cognitive assessment battery) could identify cognitive impairment in individuals seeking healthcare for stress-related exhaustion.
Ellbin[81]	2021	Cross-sectional, case control	ED 51;<br>Recovered ED 98; Control 50	74	Investigate self-reported cognitive difficulties, daily life activities, and health/sleep factors in former ED patients who still fulfill the clinical criteria for ED 7–12 years after seeking care.
Jonsdottir[82]	2013	Cross-sectional, case control	ED 33;<br>Control 37	77	To compare cognitive function in individuals with ED and healthy controls and explore if neuropsychological findings were related to severity of illness.
Jonsdottir[83]	2017	Longitudinal; cross-sectional, case control	ED 30;<br>Control 27	60	Examine if cognitive impairment is still present in patients with ED 2–3 years after seeking care.
Krabbe[84]	2017	Cross-sectional, case control	ED 25;<br>Control 25	72	Explore perceived fatigue and the effects of distraction when performing executive and complex attentional tasks.

Nelson[85]	2021	Cross-sectional, case control	ED 103; Control 58	84	Examine how individuals with ED differ from healthy controls with regard to levels and type of subjective cognitive complaints, and if such complaints are associated with cognitive test performance and psychological distress.
Österberg[86]	2009	Cross-sectional, case control	ED 65; Control 65	71	Explore cognitive problems in ED and the associations between subjective and objective cognitive performance and diurnal cortisol pattern and the DST response.
Österberg[87]	2012	Longitudinal, cohort	45	71	Determine if recovery from burnout is associated with improved cognitive functioning and if improvement is associated with changes in HPA axis activity and return to work rates.
Österberg[88]	2014	Cross-sectional, case control	Former-ED 54; Control 50	73	Assess long-term cognitive performance after substantial recovery from ED in relation to subjective cognitive complaints and return to active work.
Biological measures, n = 24					
Ekstedt[89]	2009	Longitudinal, case control	ED 23; Control 16	72	Investigate the role of sleep physiology in recovery from burnout/ED and the relation between sleep and changes in fatigue and return to work.
Hadrevi[90]	2019	Cross-sectional, case control	ED 20; Control 21	54	Investigate metabolic functions in individuals diagnosed with ED and to compare them with healthy controls.
Jónsdóttir[91]	2009	Cross-sectional, case control	Part 1: ED 42; Control 42 Part 2: ED 89; Control 88	Part 1: 100 Part 2: 54	Confirm potential biomarkers of prolonged psychosocial stress in female ED participants suggested in a former study: monocyte chemotactic protein-1 (MCP-1), epidermal growth factor (EGF) and vascular endothelial growth factor (VEGF).
Jönsson[92]	2015	Test-retest, case control	Former ED 14; Pre-ED 17; Control 20	51	Examine whether dysfunctional flexibility of the stress response in the hypothalamus-pituitary-adrenal (HPA) axis and the sympathetic-adreno-medullar (SAM) axes is present during early stages of ED, and still present after recovery.
Lennartsson[93]	2015	Cross-sectional, case control	ED 17; Control 13	37	Investigate the DHEA-s response during acute psychosocial stress in individuals with ED.
Lennartsson[94]	2015	Cross-sectional, case control	ED 19; Control 37	66	Investigate whether individuals with ED exhibit aberrant cortisol and ACTH responses to acute psychosocial stress compared with healthy individuals.
Lennartsson[95]	2015	Cross-sectional, case control	ED 122; Control 47	49	Investigate DHEA-s levels in individuals with ED compared to healthy controls.
Lennartsson[96]	2016	Longitudinal, cohort	122	49	Investigate possible changes in DHEA-S levels in ED participants and examine whether these changes are associated with health-development.
Lennartsson[97]	2016[†]	Cross-sectional, case control	ED 54; Non-clinical burnout 52; Control 55	60	To investigate heart rate variability in individuals with ED compared to individuals with non-clinical burnout and to healthy controls.

Lindegård[98]	2019	Longitudinal, cohort	88	100	Investigate longitudinal associations between cardiorespiratory fitness and self-reported physical activity levels and the severity of symptoms connected to ED, depression, anxiety, and sleep disturbances among women clinically diagnosed with ED.
Malmberg Gavelin[99]	2017	Cross-sectional, case control; longitudinal	Baseline 55; Follow-up:10; Control 11	Baseline: 84 Follow-up: 49	Investigate the association between the key symptom of ED and functional neural response during working-memory processing. Additionally, neural effects of cognitive training (CT) as part of stress rehabilitation were investigated.
Malmberg Gavelin[100]	2020	Cross-sectional	55	84	Investigate cortical and subcortical structural neural correlates of mental fatigue in individuals with ED, and to explore the association between mental fatigue and cognitive functioning.
Olsson[101]	2010	Cross-sectional, case control	ED 36; Control 19	100	Investigate possible differences between women with stress-related fatigue/ED and healthy women in heart rate variability (HRV) and other autonomic and respiratory measures, task performance and in salivary cortisol response.
Savic[102]	2018	Cross-sectional; Longitudinal, case control	ED 48; Control 80, Follow-up: ED 25; Control 19	Baseline: 59 Follow-up: 61	Investigate the cerebral effects of chronic occupational stress and its possible reversibility.
Savic[103]	2020	Cross-sectional, case control	ED 30; Control 31	51	Investigate regional glutamate concentrations using single-voxel MR spectroscopy (MRS) in participants with ED.
Sjörs[104]	2012	Longitudinal, case control	ED 162; Control 79	59	Investigate differences in HPA axis activity between individuals with ED and healthy controls and to investigate longitudinal changes in HPA axis activity in the ED group during multimodal rehabilitation.
Sjörs[105]	2013	Cross-sectional, case control	ED 90; Control 90	51	Test the usability of the allostatic load (AL) index for a clinical population with severe ED. Hypothesis was that AL would be greater in ED sample.
Sjörs[106]	2015	Longitudinal, case control	ED 122; Control 98	66	Investigate possible deviations in the diurnal cortisol profiles of individuals with clinically diagnosed ED compared with healthy controls.
Sjörs[107]	2019	Cross-sectional, case control	ED 40; Control 40	50	Investigate if circulating levels of EGF, VEGF and BDNF were altered in individuals with ED and if the level of these factors were related to symptom duration and severity in the ED group.
Skau[108]	2021	Test-retest, case control	ED 20; Control 20	70	Investigate cognitive performance and functional activity in the PFC during prolonged mental activity in individuals with ED vs healthy controls.
Sonntag-Öström[109]	2014	Repeated measures, experimental	20	100	Investigated differences in perceived restorativeness, mood, attention capacity and physiological reactions when visiting city and forest environments.
Wallensten[110]	2016	Longitudinal, case control	ED 105; Control 116	100	Examine the role of VEGF, EGF and MCP-1 in women with ED and at least 50% sick-leave and healthy women during a follow-up period of two years.

Wallensten[111]	2021	Cross-sectional, case control	ED 31; MDD 31; Control 61	85	Examine if astrocyte-derived extracellular vesicles (EV) exist in the peripheral blood of individuals with ED and if concentrations of EVs differ between ED, individuals with major depressive disorder (MDD), and healthy controls.
Wallensten[112]	2021	Cross-sectional, case control	ED 31; MDD 31; Control 561	85	To compare plasma levels of different isoforms of VEGF, including VEGF121, VEGF165, and VEGF121+VEGF165 (VEGFtotal) in individuals with ED, patients with major depressive disorder (MDD), and healthy controls.
Symptom measurement scales, n = 4					
Axelsson[113]	2017[†]	Longitudinal	160	78	To study the psychometric properties of the 12-item self-report WHODAS 2.0 when administered online to individuals with anxiety and stress disorders.
Beser[114]	2014	Cross-sectional, case control	ED 200; Control 117	80	To construct and evaluate a self-rating scale, the Karolinska Exhaustion Disorder Scale (KEDS), for the assessment of ED symptoms.
Lundgren-Nilsson[115]	2012	Cross-sectional, case control	ED 319; Control 319	69	To examine the properties of the Shirom-Melamed Burnout Questionnaire (SMBQ) for validation of use in a clinical setting.
Lundgren-Nilsson[116]	2013	Longitudinal	179	70	To evaluate the Psychological general well-being index (PGWBI) with Rasch- and factor analysis.
Interventions, n = 29					
Adevi[117]	2012	Qualitative	5	80	Explore caregiver perspective on factors considered most essential to the recovery process of patients with ED.
Adevi[118]	2013	Qualitative	5	80	Explore the impact of garden therapy on stress-rehabilitation with a special focus on nature.
Eskilsson[40]	2017	RCT[‡]	MMR+AT 47; MMR 41	88	Investigate the effects on cognitive performance and psychological variables of a 12-week aerobic training (AT) program performed at a moderate-vigorous intensity for individuals with exhaustion disorder who participated in a multimodal rehabilitation program (MMR)l.
Eskilsson[119]	2020	Qualitative	13	85	Explore experiences from persons with ED after participating in a 12-week intervention of MMR with either additional computerized cognitive training or aerobic training.
Finnes[120]	2017[†]	RCT	ACT 89; WDI 87; ACT+WDI 88; TAU 88	78	Evaluate the efficacy of 3 interventions targeting sickness absence of workers. Randomization to (a) acceptance and commitment therapy (ACT), (b) a workplace dialogue intervention (WDI), (c) a combination of ACT and WDI, or (d) treatment as usual (TAU).
Finnes[121]	2017[†]	RCT, secondary analysis	ACT 89; WDI 87; ACT+WDI 88; TAU 88	78	To evaluate cost-utility of ACT and WDI, both as stand-alone interventions and in combination, compared with treatment as usual (TAU), for employees on sickness absence with mental disorders.

Gerber[42]	2015	Longitudinal, cohort	Exercise 36; General advice 133	79	Examine the changes in exercise habits during a 12-month MMR treatment and possible differences between general exercise instructions and an additional 18-week coached exercise program.
Grensman[43]	2018	RCT	TY 32; MBCT 31; CBT 31	89	Assess the effects of a long (20 weeks) treatment with traditional yoga (TY), mindfulness-based cognitive behavioral therapy (MBCT) and cognitive behavioral therapy (CBT; active control) on health-related quality of live (HRQoL) in individuals with ED on sick leave.
Karlson[44]	2010[†]	Longitudinal, case control	CDM 74; Control 74	78	Evaluate the effect of a “convergence dialogue meeting”-intervention (CDM; job-person match through patient-supervisor communication) with individuals being treated for burnout compared to a waitlist control of individuals not wanting the CDM-intervention.
Karlson[122]	2014[†]	Longitudinal, case control	CDM 68; Control 68	81	Whether the effects of CDM-intervention (presented in Karlson, 2010) were sustained or increased further during an additional 12 months, or whether the intervention merely speeded up the course of return to work.
Lindegård[45]	2015	Longitudinal, cohort	69	65	Investigate whether initially physically inactive individuals diagnosed with ED differ at the 6-month, 12-month and 18-month follow-up regarding burnout (as a primary outcome), depressive symptoms, and anxiety symptoms depending on whether they (mildly or strongly) complied or did not comply with the physical activity recommendations.
Lindsäter[123]	2018[†]	RCT	ICBT 50; Waitlist 50	85	To investigate the efficacy of internet-delivered cognitive behavioral therapy (ICBT) for individuals suffering from chronic stress, operationalized as adjustment disorder and ED.
Lindsäter[124]	2019[†]	RCT, secondary analysis	ICBT 50; Waitlist 50	85	Evaluate the cost-effectiveness and cost-utility of ICBT for individuals with stress-related disorders in the form of adjustment disorder or ED.
Lindsäter[125]	2021[†]	RCT, secondary analysis	ICBT 50; Waitlist 50	85	To investigate insomnia symptom severity as a putative mediator of treatment response in internet-based CBT for chronic stress, using data from a randomized controlled trial.
Malmberg Gavelin[47]	2015	RCT[‡]	MMR + CT 27; MMR 32	85	Evaluate the effects of Multimodal rehabilitation (MMR) + a cognitive training (CT) intervention in comparison with only MMR on cognitive performance and subjective cognitive complaints.
Malmberg Gavelin[48]	2018	RCT[‡]	MMR+CT 44; MMR+AT 47; MMR 41	84	Investigate the long-term effects (1 year follow-up) of 12 weeks cognitive (CT) or aerobic (AT) training on cognitive function, psychological health, and work ability for individuals diagnosed with ED.
Millet[49]	2008	Pretest-posttest, pilot	32	100	To examine whether gardening therapy could be a possible method to be used in the vocational rehabilitation of persons sick-listed due to high levels of stress.
Nordh[50]	2009[†]	Mixed method	24	50	If individuals with ED on long-term sick-leave can gain improved health when undertaking meaningful activities in a forest (forest-therapy).
Olsson[51]	2009	RCT	R. Rosea 30; Placebo 30	90	To determine whether the daily intake of R. rosea extract SHR-5 over a 28-day period would produce any positive effects on attention, quality of life, and symptoms of fatigue and depression in subjects with stress-related fatigue.

Ristiniemi[52]	2014	Pretest-posttest, case control	African dance 15; ED control 15; Healthy controls 14	82	To systematically study the role of disturbed breathing in individuals with ED and to explore the efficacy of utilizing the African dance of Grounding as a type of short-term physical therapy for normalizing their respiratory patterns.
Salomonsson[126]	2017[†]	RCT	CBT 64; RTW-I 67; COMBO 80	82	To evaluate CBT, a return-to-work intervention (RTW-I) and combined CBT and RTW-I (COMBO) for primary care patients on sick leave due to common mental disorders.
Salomonsson[127]	2020[†]	RCT, subgroup analysis	Stress 152; Control 59	82	To evaluate the effect of CBT for stress-related disorders (adjustment disorder or ED) and explore whether RTW-I, alone or in combination with disorder specific CBT, has different effects for individuals diagnosed with stress-related disorders than for individuals with other primary common mental disorders (depression, anxiety, insomnia).
Santoft[128]	2019	RCT, secondary analysis	CBT 40; RTW-I 42	84	To investigate potential mediators of change for individuals with ED receiving CBT, compared to a return-to-work intervention (RTW-I).
Sonntag-Öström[129]	2011	Mixed method, pilot	6	50	To examine whether the boreal forest in northern Sweden can be used for rehabilitation from stress-related exhaustion.
Sonntag-Öström[54]	2015	RCT	Forest 51; Control 48	86	To evaluate if participation in the forest intervention can enhance recovery from ED in comparison with the wait-list control group.
Sonntag-Öström[130]	2015	Qualitative	19	84	Investigate the personal experiences and perceived effects from visits to forest environments in a subset of individuals with severe ED.
Stenlund[55]	2009	RCT	Qigong 41; Control 41	83	Evaluate the effectiveness of "basic care" + a biweekly 12-week Qigong intervention in comparison with only basic care (control condition) in individuals with ED.
Strömbäck[131]	2020	Qualitative	15	87	Investigate experiences from a patient perspective of a dialogue-based workplace intervention with convergence dialogue meetings that was performed by a rehabilitation coordinator.
van de Leur[132]	2020	Longitudinal, cohort	390	88	Explore changes in ED symptoms and return-to-work-rates in individuals with ED participating in a standardized MMR in a clinical setting.

[†]Recruitment of blended samples of which ED was a subsample

[‡]Same RCT reporting on different comparison groups and outcomes

Table S5. Overview of dissertations (*N* = 17) for doctoral degree that entail studies on exhaustion disorder.

**Author, year**	**Study location**	**Title of thesis**	**Aim**
Engebretsen, 2020	Oslo, Norway	From dedicated to burned out - and back? A phenomenological exploration of the lived experience of suffering from burnout and implications for medical care	To explore the lived experience of burnout with special attention to the factors they experience as enhancing or restricting their rehabilitation process.
Eriksson, 2016	Sundsvall, Sweden	(In Swedish[†]) At the point of exhaustion. Clinical burn-out as an existential state. Health care providers' and patients' experiences of clinical burn-out and rehabilitation with an existential approach in the Swedish health care context.	To gain insight into the existential experience of clinical burn-out as well as to highlight the significance of an existential perspective in rehabilitation.
Finnes, 2018	Stockholm, Sweden	Return to work - methods for promoting health and productivity in employees on sickness absence	To evaluate the effect of psychological interventions on sickness absence and return to work.
Glise, 2014	Gothenburg, Sweden	Exhaustion disorder - identification, characterization, and course of illness	To study exhaustion disorder (ED) with respect to identification, characterization, and course of illness. Additionally, to study properties of an instrument of self-rated ED (s-ED).
Grensman, 2020	Stockholm, Sweden	Traditional yoga and clinical burnout - quality of life and biomarkers before and after treatment	To understand the situation of patients with clinical burnout (CB) on sick leave, to investigate the effect of traditional yoga (TY) on CB, and whether there are subjective and objective measures that can be used for screening to diagnose CB, to follow the course, and evaluate treatment effects.
Gustafsson, 2009	Umeå, Sweden	(In Swedish[†]) To become or not to become burned out - a complex phenomenon among healthcare professionals in the same workplaces*	To describe the meaning of becoming burnt out and describe personality traits and views on conscience, stress, moral and social support among ED and non-ED participants.
Lindsäter, 2020	Stockholm, Sweden	Cognitive behavioral therapy for stress-related disorders	To build and expand on the limited knowledge base regarding CBT as a treatment for stress-related disorders by further investigating clinical efficacy, cost-effectiveness, and mediators of change in treatment.

Malmberg Gavelin, 2019	Umeå, Sweden	Rehabilitation for improved cognition in stress-related exhaustion - cognitive neural and clinical perspectives	To evaluate the efficacy of additional cognitive and aerobic training for patients with ED who participated in a multimodal stress rehabilitation program and to explore the neural correlates of ED.
Norlund, 2011	Umeå, Sweden	Psychosocial work factors and burnout - a study of a working general population and patients at a stress rehabilitation clinic	To assess the level of burnout in a working general population and investigate the importance of psychosocial work factors and sex on burnout and to study reduction of sick leave and experiences of returning to work in burnout patients, with special attention towards psychosocial work factors.
Persson Asplund, 2021	Linköping, Sweden	Learning how to recover from stress-related disorders via internet-based interventions	To bring further evidence to the field on the experiences and efficacy of internet-based and work-focused interventions for employees with stress-related disorders.
Sahlin, 2014	Alnarp, Sweden	To stress the importance of nature - nature-based therapy for the rehabilitation and prevention of stress-related disorders	To explore whether Nature-Based Therapy (NBT) for prevention and rehabilitation positively affected participants' health and well-being; that is, their physical and mental health and well-being, as well as their ability to function in everyday life.
Salomonsson, 2018	Stockholm, Sweden	CBT in primary care - effects on symptoms and sick leave, implementation of stepped care and predictors of outcome	To implement and evaluate evidence-based CBT, to evaluate CBT for adjustment and exhaustion disorders, and to evaluate an intervention to reduce sick leave (RTW-I) among patients with common mental disorders in primary care.
Santoft, 2019	Stockholm, Sweden	What makes cognitive behavior therapy work? An investigation of psychological and inflammatory processes	To investigate processes and correlates of therapeutic change in CBT for common mental disorders.
Sonntag-Öström, 2014	Umeå, Sweden	Forest for rest - recovery from exhaustion disorder	To study whether visits to different kinds of forest environments have positive health effects on patients suffering from ED and can be utilized for rehabilitation.
Stenlund, 2009	Umeå, Sweden	Rehabilitation for patients with burnout	To describe patients on long-term sick leave because of burnout and to evaluate rehabilitation programs for this patient group.

Söderström, 2012	Stockholm, Sweden	Burnout - a matter of impaired recovery?	To investigate physiological and subjective markers of recovery from stress to identify and discuss possible risk factors precipitating burnout, as well as factors related to recovery from burnout and return to work. Sleep and unwinding during leisure time were in particular focus.
Wahlberg, 2012	Stockholm, Sweden	Stress reactivity, cognitive functioning and hippocampal morphology in exhaustion disorder, and development of a self-rating scale for exhaustion disorder, KEDS	To obtain insights into the biological process associated with work stress related depression and exhaustion disorder in women, and to construct and evaluate a self-rating scale for assessment of symptoms of exhaustion disorder.

†Translation to English by research team

References

1. Gustafsson G, Norberg A, Strandberg G. Meanings of becoming and being burnout--phenomenological-hermeneutic interpretation of female healthcare personnel's narratives. *Scand J Caring Sci*. 2008; **22**: 520-8.
2. Håkansson C, Matuska KM. How life balance is perceived by Swedish women recovering from a stress-related disorder: A validation of the life balance model. *Journal of Occupational Science*. 2010; **17**: 112-9.
3. Asplund S, Åhlin J, Åström S, Hedlund M, Lindgren BM, Ericson-Lidman E. Self-rated exhaustion disorder and associated health-related factors among municipal employees in rural areas of northern Sweden. *Int Arch Occup Environ Health*. 2021; **94**: 659-68.
4. Broddadóttir E, Flóvenz S, Gylfason HF, Þormar Þ, Einarsson H, Salkovskis P, et al. "I'm So Tired": Fatigue as a Persistent Physical Symptom among Working People Experiencing Exhaustion Disorder. *Int J Environ Res Public Health*. 2021; **18**.
5. Gustafsson G, Persson B, Eriksson S, Norberg A, Strandberg G. Personality traits among burnt out and non-burnt out health-care personnel at the same workplaces: a pilot study. *Int J Ment Health Nurs*. 2009; **18**: 336-48.
6. Gustafsson G, Eriksson S, Strandberg G, Norberg A. Burnout and perceptions of conscience among health care personnel: a pilot study. *Nurs Ethics*. 2010; **17**: 23-38.
7. Håkansson C, Ahlborg G, Jr. Occupational imbalance and the role of perceived stress in predicting stress-related disorders. *Scand J Occup Ther*. 2018; **25**: 278-87.
8. Höglund P, Hakelind C, Nordin S. Severity and prevalence of various types of mental ill-health in a general adult population: age and sex differences. *BMC Psychiatry*. 2020; **20**: 209.
9. Norlund S, Reuterwall C, Hoog J, Nordin M, Edlund C, Birgander LS. Work Related Factors and Sick Leave After Rehabilitation in Burnout Patients: Experiences from the REST-Project. *Journal of Occupational Rehabilitation*. 2011; **21**: 23-30.
10. Stenlund T, Ahlgren C, Lindahl B, Burell G, Knutsson A, Stegmayr B, et al. Patients with burnout in relation to gender and a general population. *Scand J Public Health*. 2007; **35**: 516-23.
11. Soderstrom M, Jeding K, Ekstedt M, Perski A, Akerstedt T. Insufficient sleep predicts clinical burnout. *J Occup Health Psychol*. 2012; **17**: 175-83.
12. Wiegner L, Hange D, Björkelund C, Ahlborg G. Prevalence of perceived stress and associations to symptoms of exhaustion, depression and anxiety in a working age population seeking primary care-an observational study. *BMC family practice*. 2015; **16**: 1.
13. Sandstrom A, Rhodin IN, Lundberg M, Olsson T, Nyberg L. Impaired cognitive performance in patients with chronic burnout syndrome. *Biol Psychol*. 2005; **69**: 271-9.
14. Blix E, Perski A, Berglund H, Savic I. Long-term occupational stress is associated with regional reductions in brain tissue volumes. *PLoS One*. 2013; **8**: e64065.
15. Bäckström T, Bixo M, Nyberg S, Savic I. Increased neurosteroid sensitivity--an explanation to symptoms associated with chronic work related stress in women? *Psychoneuroendocrinology*. 2013; **38**: 1078-89.
16. Ekstedt M, Söderström M, Åkerstedt T, Nilsson J, Søndergaard H-P, Perski A. Disturbed sleep and fatigue in occupational burnout. *Scand J Work Environ Health*. 2006; **32**: 121-31.
17. Golkar A, Johansson E, Kasahara M, Osika W, Perski A, Savic I. The influence of work-related chronic stress on the regulation of emotion and on functional connectivity in the brain. *PLoS One*. 2014; **9**: e104550.
18. Grossi G, Perski A, Ekstedt M, Johansson T, Lindström M, Holm K. The morning salivary cortisol response in burnout. *J Psychosom Res*. 2005; **59**.
19. Heiden M, Barnekow-Bergkvist M, Nakata M, Lyskov E. Autonomic activity, pain, and perceived health in patients on sick leave due to stress-related illnesses. *Integr Physiol Behav Sci*. 2005; **40**: 3-16.
20. Jovanovic H, Perski A, Berglund H, Savic I. Chronic stress is linked to 5-HT(1A) receptor changes and functional disintegration of the limbic networks. *Neuroimage*. 2011; **55**: 1178-88.

21. Sandstrom A, Peterson J, Sandstrom E, Lundberg M, Nystrom IL, Nyberg L, et al. Cognitive deficits in relation to personality type and hypothalamic-pituitary-adrenal (HPA) axis dysfunction in women with stress-related exhaustion. *Scand J Psychol*. 2011; **52**: 71-82.
22. Sandström A, Säll R, Peterson J, Salami A, Larsson A, Olsson T, et al. Brain activation patterns in major depressive disorder and work stress-related long-term sick leave among Swedish females. *Stress*. 2012; **15**: 503-13.
23. Savic I. Structural changes of the brain in relation to occupational stress. *Cereb Cortex*. 2015; **25**: 1554-64.
24. Glise K, Hadzibajramovic E, Jonsdottir I, Ahlborg G. Self-reported exhaustion: a possible indicator of reduced work ability and increased risk of sickness absence among human service workers. *International archives of occupational and environmental health*. 2010; **83**: 511-20.
25. Persson R, Österberg K, Viborg N, Jönsson P, Tenenbaum A. The Lund University Checklist for Incipient Exhaustion–a cross–sectional comparison of a new instrument with similar contemporary tools. *BMC public health*. 2016; **16**: 350.
26. Persson R, Österberg K, Viborg N, Jönsson P, Tenenbaum A. Two Swedish screening instruments for exhaustion disorder: cross-sectional associations with burnout, work stress, private life stress, and personality traits. *Scandinavian journal of public health*. 2017; **45**: 381-8.
27. Saboonchi F, Perski A, Grossi G. Validation of Karolinska Exhaustion Scale: psychometric properties of a measure of exhaustion syndrome. *Scand J Caring Sci*. 2013; **27**: 1010-7.
28. Cerwén G, Pedersen E, Pálsdóttir AM. The Role of Soundscape in Nature-Based Rehabilitation: A Patient Perspective. *Int J Environ Res Public Health*. 2016; **13**.
29. Fjellman-Wiklund A, Stenlund T, Steinholtz K, Ahlgren C. Take charge: Patients' experiences during participation in a rehabilitation programme for burnout. *J Rehabil Med*. 2010; **42**: 475-81.
30. Grahn P, Palsdottir AM, Ottosson J, Jonsdottir IH. Longer Nature-Based Rehabilitation May Contribute to a Faster Return to Work in Patients with Reactions to Severe Stress and/or Depression. *Int J Environ Res Public Health*. 2017; **14**.
31. Grossi G, Santell B. Quasi-experimental evaluation of a stress management programme for female county and municipal employees on long-term sick leave due to work-related psychological complaints. *J Rehabil Med*. 2009; **41**: 632-8.
32. Heiden M, Lyskov E, Nakata M, Sahlin K, Sahlin T, Barnekow-Bergkvist M. Evaluation of cognitive behavioural training and physical activity for patients with stress-related illnesses: a randomized controlled study. *J Rehabil Med*. 2007; **39**: 366-73.
33. Nygren A, Nasman P, Ekerlund L, Kjellstrom B, Lofman B. [Residential multimodal job focused rehabilitation for teachers increases return to work]. *Internatbehandling hjalper larare med utmattningssyndrom tillbaka i arbete - En kontrollerad studie av rehabilitering i internatform*. 2019; **116**.
34. Pálsdóttir AM, Grahn P, Persson D. Changes in experienced value of everyday occupations after nature-based vocational rehabilitation. *Scand J Occup Ther*. 2014; **21**: 58-68.
35. Pálsdóttir AM, Persson D, Persson B, Grahn P. The journey of recovery and empowerment embraced by nature - clients' perspectives on nature-based rehabilitation in relation to the role of the natural environment. *Int J Environ Res Public Health*. 2014; **11**: 7094-115.
36. Persson Asplund R, Dagöö J, Fjellstrom I, Niemi L, Hansson K, Zeraati F, et al. Internet-based stress management for distressed managers: results from a randomised controlled trial. *Occup Environ Med*. 2018; **75**: 105-13.
37. Sahlin E, Ahlborg G, Jr., Tenenbaum A, Grahn P. Using nature-based rehabilitation to restart a stalled process of rehabilitation in individuals with stress-related mental illness. *Int J Environ Res Public Health*. 2015; **12**: 1928-51.
38. Stenlund T, Ahlgren C, Lindahl B, Burell G, Steinholtz K, Edlund C, et al. Cognitively oriented behavioral rehabilitation in combination with Qigong for patients on long-term sick leave because of burnout: REST--a randomized clinical trial. *Int J Behav Med*. 2009; **16**: 294-303.

39. Stenlund T, Nordin M, Jarvholm LS. Effects of rehabilitation programmes for patients on long-term sick leave for burnout: a 3-year follow-up of the REST study. *J Rehabil Med*. 2012; **44**: 684-90.
40. Eskilsson T, Slunga Jarvholm L, Malmberg Gavelin H, Stigsdotter Neely A, Boraxbekk CJ. Aerobic training for improved memory in patients with stress-related exhaustion: a randomized controlled trial. *BMC Psychiatry*. 2017; **17**: 322.
41. Finnes A, Ghaderi A, Dahl J, Nager A, Enebrink P. Randomized controlled trial of acceptance and commitment therapy and a workplace intervention for sickness absence due to mental disorders. *J Occup Health Psychol*. 2019; **24**: 198-212.
42. Gerber M, Jonsdottir IH, Arvidson E, Lindwall M, Lindegård A. Promoting graded exercise as a part of multimodal treatment in patients diagnosed with stress-related exhaustion. *Journal of clinical nursing*. 2015; **24**: 1904-15.
43. Grensman A, Acharya BD, Wandell P, Nilsson GH, Falkenberg T, Sundin O, et al. Effect of traditional yoga, mindfulness-based cognitive therapy, and cognitive behavioral therapy, on health related quality of life: a randomized controlled trial on patients on sick leave because of burnout. *BMC complementary and alternative medicine*. 2018; **18**: 80.
44. Karlson B, Jonsson P, Palsson B, Abjornsson G, Malmberg B, Larsson B, et al. Return to work after a workplace-oriented intervention for patients on sick-leave for burnout--a prospective controlled study. *BMC Public Health*. 2010; **10**: 301.
45. Lindegård A, Jonsdottir IH, Börjesson M, Lindwall M, Gerber M. Changes in mental health in compliers and non-compliers with physical activity recommendations in patients with stress-related exhaustion. *BMC psychiatry*. 2015; **15**: 272-.
46. Lindsäter E, Axelsson E, Salomonsson S, Santoft F, Ejeby K, Ljotsson B, et al. Internet-Based Cognitive Behavioral Therapy for Chronic Stress: A Randomized Controlled Trial. *Psychother Psychosom*. 2018; **87**: 296-305.
47. Malmberg Gavelin H, Boraxbekk C-J, Stenlund T, Jarvholm LS, Neely AS. Effects of a process-based cognitive training intervention for patients with stress-related exhaustion. *Stress (Amsterdam, Netherlands)*. 2015: 1-11.
48. Malmberg Gavelin H, Eskilsson T, Boraxbekk CJ, Josefsson M, Stigsdotter Neely A, Slunga Järvholm L. Rehabilitation for improved cognition in patients with stress-related exhaustion disorder: RECO - a randomized clinical trial. *Stress*. 2018; **21**: 279-91.
49. Millet P. Integrating Horticulture into the Vocational Rehabilitation Process of Individuals with Exhaustion Syndrome (Burnout): A Pilot Study. 392009.
50. Nordh H, Grahn P, Währborg P. Meaningful activities in the forest, a way back from exhaustion and long-term sick leave. *Urban forestry & urban greening*. 2009; **8**: 207-19.
51. Olsson EM, von Schéele B, Panossian AG. A randomised, double-blind, placebo-controlled, parallel-group study of the standardised extract shr-5 of the roots of Rhodiola rosea in the treatment of subjects with stress-related fatigue. *Planta medica*. 2009; **75**: 105-12.
52. Ristiniemi H, Perski A, Lyskov E, Emtner M. Hyperventilation and exhaustion syndrome. *Scandinavian journal of caring sciences*. 2014; **28**: 657-64.
53. Salomonsson S, Santoft F, Lindsater E, Ejeby K, Ljotsson B, Ost LG, et al. Cognitive-behavioural therapy and return-to-work intervention for patients on sick leave due to common mental disorders: a randomised controlled trial. *Occup Environ Med*. 2017; **74**: 905-12.
54. Sonntag-Öström E, Nordin M, Dolling A, Lundell Y, Nilsson L, Slunga Järvholm L. Can rehabilitation in boreal forests help recovery from exhaustion disorder? The randomised clinical trial ForRest. *Scandinavian journal of forest research*. 2015; **30**: 732-48.
55. Stenlund T, Birgander LS, Lindahl B, Nilsson L, Ahlgren C. EFFECTS OF QIGONG IN PATIENTS WITH BURNOUT: A RANDOMIZED CONTROLLED TRIAL. *Journal of Rehabilitation Medicine*. 2009; **41**: 761-7.
56. van de Leur JC, Buhrman M, Åhs F, Rozental A, Jansen GB. Standardized multimodal intervention for stress-induced exhaustion disorder: an open trial in a clinical setting. *BMC psychiatry*. 2020; **20**: 1-14.
57. Alsén S, Ali L, Ekman I, Fors A. Facing a blind alley-Experiences of stress-related exhaustion: a qualitative study. *BMJ open*. 2020; **10**: e038230.

58. Arman M, Hammarqvist A-S, Rehnsfeldt A. Burnout as an existential deficiency - lived experiences of burnout sufferers. *Scandinavian journal of caring sciences*. 2011; **25**: 294-302.
59. Engebretsen KM. Suffering without a medical diagnosis. A critical view on the biomedical attitudes towards persons suffering from burnout and the implications for medical care. *Journal of evaluation in clinical practice*. 2018; **24**: 1150-7.
60. Engebretsen KM, Bjorbaekmo WS. Naked in the eyes of the public: A phenomenological study of the lived experience of suffering from burnout while waiting for recognition to be ill. *Journal of evaluation in clinical practice*. 2019; **25**: 1017-26.
61. Engebretsen KM, Bjorbækmo WS. Out of Chaos—Meaning Arises: The Lived Experience of Re-Habituating the Habitual Body When Suffering From Burnout. *Qualitative health research*. 2020; **30**: 1468-79.
62. Ericson-Lidman E, Strandberg G. Burnout: co-workers' perceptions of signs preceding workmates' burnout. *Journal of Advanced Nursing*. 2007; **60**: 199-208.
63. Hörberg U, Wagman P, Gunnarsson AB. Women's lived experience of well-being in everyday life when living with a stress-related illness. *International journal of qualitative studies on health and well-being*. 2020; **15**: 1754087-.
64. Jingrot M, Rosberg S. Gradual loss of homelikeness in exhaustion disorder. *Qual Health Res*. 2008; **18**: 1511-23.
65. Norlund S, Fjellman-Wiklund A, Nordin M, Stenlund T, Ahlgren C. Personal resources and support when regaining the ability to work: an interview study with Exhaustion Disorder patients. *J Occup Rehabil*. 2013; **23**: 270-9.
66. Adamsson A, Bernhardsson S. Symptoms that may be stress-related and lead to exhaustion disorder: a retrospective medical chart review in Swedish primary care. *BMC family practice*. 2018; **19**: 172.
67. Beno A, Hensing G, Lindegård A, Jonsdottir IH. Self-reported changes in work situation – a cross-sectional study of patients 7 years after treatment for stress-related exhaustion. *BMC public health*. 2021; **21**: 1222-.
68. Glise K, Ahlborg G, Jr., Jonsdottir IH. Course of mental symptoms in patients with stress-related exhaustion: does sex or age make a difference? *BMC Psychiatry*. 2012; **12**.
69. Glise K, Ahlborg G, Jonsdottir IH. Prevalence and course of somatic symptoms in patients with stress-related exhaustion: does sex or age matter. *BMC psychiatry*. 2014; **14**.
70. Glise K, Wiegner L, Jonsdottir IH. Long-term follow-up of residual symptoms in patients treated for stress-related exhaustion. *BMC psychology*. 2020; **8**: 26.
71. Grensman A, Acharya BD, Wandell P, Nilsson G, Werner S. Health-related quality of life in patients with Burnout on sick leave: descriptive and comparative results from a clinical study. *Int Arch Occup Environ Health*. 2016; **89**: 319-29.
72. Grossi G, Jeding K, Soderstrom M, Osika W, Levander M, Perski A. Self-reported sleep lengths >/= 9 hours among Swedish patients with stress-related exhaustion: Associations with depression, quality of sleep and levels of fatigue. *Nord J Psychiatry*. 2015; **69**: 292-9.
73. Grossi G, Jeding K, Soderstrom M, Perski A, Alfoldi P, Osika W. Alcohol Use among Swedish Patients with Stress-Induced Exhaustion Disorder, and Its Relation to Anxiety, Depression, and Health-Related Quality of Life. *International journal of mental health and addiction*. 2021.
74. Gulin S, Ellbin S, Jonsdottir IH, Lindqvist Bagge AS. Is obsessive–compulsive personality disorder related to stress-related exhaustion? *Brain and behavior*. 2021; **11**: e02171-n/a.
75. Hasselberg K, Jonsdottir IH, Ellbin S, Skagert K. Self-reported stressors among patients with exhaustion disorder: an exploratory study of patient records. *BMC Psychiatry*. 2014; **14**: 66.
76. Maroti D, Molander P, Bileviciute-Ljungar I. Differences in alexithymia and emotional awareness in exhaustion syndrome and chronic fatigue syndrome. *Scandinavian journal of psychology*. 2017; **58**: 52-61.
77. Maroti D, Bileviciute-Ljungar I. Similarities and differences between health-related quality of life in patients with exhaustion syndrome and chronic fatigue syndrome. *Fatigue: Biomedicine, Health & Behavior*. 2018; **6**: 208-19.

78. Skoglund I, Björkelund C, Svenningsson I, Petersson E-L, Augustsson P, Nejati S, et al. Influence of antidepressant therapy on sick leave in primary care: ADAS, a comparative observational study. *Heliyon*. 2019; **5**: e01101.
79. Bartfai A, Åsberg M, Beser A, Sorjonen K, Wilczek A, Warkentin S. Impaired cognitive functioning in stress-induced exhaustion disorder: a new tablet-based assessment. *BMC psychiatry*. 2021; **21**: 459-.
80. Ellbin S, Engen N, Jonsdottir IH, Nordlund AIK. Assessment of cognitive function in patients with stress-related exhaustion using the Cognitive Assessment Battery (CAB). *Journal of clinical and experimental neuropsychology*. 2018; **40**: 567-75.
81. Ellbin S, Jonsdottir IH, Eckerström C, Eckerström M. Self-reported cognitive impairment and daily life functioning 7–12 years after seeking care for stress-related exhaustion. *Scandinavian journal of psychology*. 2021; **62**: 484-92.
82. Jonsdottir IH, Nordlund A, Ellbin S, Ljung T, Glise K, Wahrborg P, et al. Cognitive impairment in patients with stress-related exhaustion. *Stress*. 2013; **16**: 181-90.
83. Jonsdottir IH, Nordlund A, Ellbin S, Ljung T, Glise K, Währborg P, et al. Working memory and attention are still impaired after three years in patients with stress-related exhaustion. *Scandinavian Journal of Psychology*. 2017; **58**: 504-9.
84. Krabbe D, Ellbin S, Nilsson M, Jonsdottir IH, Samuelsson H. Executive function and attention in patients with stress-related exhaustion: perceived fatigue and effect of distraction. *Stress (Amsterdam, Netherlands)*. 2017; **20**: 333-40.
85. Nelson A, Gavelin HM, Boraxbekk C-J, Eskilsson T, Josefsson M, Slunga Järvholm L, et al. Subjective cognitive complaints in patients with stress-related exhaustion disorder: a cross sectional study. *BMC Psychology*. 2021; **9**: 84-.
86. Österberg K, Karlson B, Hansen ÅM. Cognitive performance in patients with burnout, in relation to diurnal salivary cortisol: Original Research Report. *Stress (Amsterdam, Netherlands)*. 2009; **12**: 70-81.
87. Österberg K, Karlson B, Malmberg B, Hansen ÅM. A follow-up of cognitive performance and diurnal salivary cortisol changes in former burnout patients. *Stress (Amsterdam, Netherlands)*. 2012; **15**: 589-600.
88. Österberg K, Skogsliden S, Karlson B. Neuropsychological sequelae of work-stress-related exhaustion. *Stress*. 2014; **17**: 59-69.
89. Ekstedt M, Söderström M, Åkerstedt T. Sleep physiology in recovery from burnout. *Biological Psychology*. 2009; **82**: 267-73.
90. Hadrevi J, Jonsdottir IH, Jansson P-A, Eriksson JW, Sjors A. Plasma metabolomic patterns in patients with exhaustion disorder. *Stress (Amsterdam, Netherlands)*. 2019; **22**: 17-26.
91. Jonsdottir IH, Hagg DA, Glise K, Ekman R. Monocyte chemotactic protein-1 (MCP-1) and growth factors called into question as markers of prolonged psychosocial stress. *PLoS One*. 2009; **4**: e7659.
92. Jönsson P, Österberg K, Wallergård M, Hansen ÅM, Garde AH, Johansson G, et al. Exhaustion-related changes in cardiovascular and cortisol reactivity to acute psychosocial stress. *Physiology & behavior*. 2015; **151**: 327-37.
93. Lennartsson A-K, Sjörs A, Jonsdottir IH. Indication of attenuated DHEA-s response during acute psychosocial stress in patients with clinical burnout. *Journal of psychosomatic research*. 2015; **79**: 107-11.
94. Lennartsson A-K, Sjörs A, Währborg P, Ljung T, Jonsdottir IH. Burnout and Hypocortisolism - A Matter of Severity? A Study on ACTH and Cortisol Responses to Acute Psychosocial Stress. *Frontiers in psychiatry*. 2015; **6**: 8-.
95. Lennartsson A-K, Theorell T, Kushnir MM, Jonsdottir IH. Low levels of dehydroepiandrosterone sulfate in younger burnout patients. *PloS one*. 2015; **10**: e0140054.
96. Lennartsson A-K, Theorell T, Kushnir MM, Jonsdottir IH. Changes in DHEA-s levels during the first year of treatment in patients with clinical burnout are related to health development. *Biological psychology*. 2016; **120**: 28-34.
97. Lennartsson A-K, Jonsdottir I, Sjörs A. Low heart rate variability in patients with clinical burnout. *International journal of psychophysiology*. 2016; **110**: 171-8.

98. Lindegard A, Wastensson G, Hadzibajramovic E, Grimby-Ekman A. Longitudinal associations between cardiorespiratory fitness and stress-related exhaustion, depression, anxiety and sleep disturbances. *BMC public health*. 2019; **19**: 1726.
99. Malmberg Gavelin H, Neely AS, Andersson M, Eskilsson T, Järvholm LS, Boraxbekk C-J. Neural activation in stress-related exhaustion: Cross-sectional observations and interventional effects. *Psychiatry research Neuroimaging*. 2017; **269**: 17-25.
100. Malmberg Gavelin H, Neely AS, Dunås T, Eskilsson T, Järvholm LS, Boraxbekk C-J. Mental fatigue in stress-related exhaustion disorder: Structural brain correlates, clinical characteristics and relations with cognitive functioning. *NeuroImage clinical*. 2020; **27**: 102337-.
101. Olsson EMG, Roth WT, Melin L. Psychophysiological characteristics of women suffering from stress-related fatigue. *Stress and Health*. 2010; **26**: 113-26.
102. Savic I, Perski A, Osika W. MRI Shows that Exhaustion Syndrome Due to Chronic Occupational Stress is Associated with Partially Reversible Cerebral Changes. *Cerebral cortex (New York, NY 1991)*. 2018; **28**: 894-906.
103. Savic I. MRS Shows Regionally Increased Glutamate Levels among Patients with Exhaustion Syndrome Due to Occupational Stress. *Cerebral cortex (New York, NY : 1991)*. 2020; **30**: 3759-70.
104. Sjörs A, Ljung T, Jonsdottir IH. Long-term follow-up of cortisol awakening response in patients treated for stress-related exhaustion. *BMJ open*. 2012; **2**: e001091.
105. Sjors A, Jansson PA, Eriksson JW, Jonsdottir IH. Increased insulin secretion and decreased glucose concentrations, but not allostatic load, are associated with stress-related exhaustion in a clinical patient population. *Stress*. 2013; **16**: 24-33.
106. Sjörs A, Jonsdottir IH. No alterations in diurnal cortisol profiles before and during the treatment in patients with stress-related exhaustion. *International journal of occupational medicine and environmental health*. 2015; **28**: 120.
107. Sjors Dahlman A, Blennow K, Zetterberg H, Glise K, Jonsdottir IH. Growth factors and neurotrophins in patients with stress-related exhaustion disorder. *Psychoneuroendocrinology*. 2019; **109**: 104415.
108. Skau S, Jonsdottir IH, Sjörs Dahlman A, Johansson B, Kuhn HG. Exhaustion disorder and altered brain activity in frontal cortex detected with fNIRS. *Stress (Amsterdam, Netherlands)*. 2021; **24**: 64-75.
109. Sonntag-Öström E, Nordin M, Lundell Y, Dolling A, Wiklund U, Karlsson M, et al. Restorative effects of visits to urban and forest environments in patients with exhaustion disorder. *Urban forestry & urban greening*. 2014; **13**: 344-54.
110. Wallensten J, Asberg M, Nygren A, Szulkin R, Wallen H, Mobarrez F, et al. Possible Biomarkers of Chronic Stress Induced Exhaustion - A Longitudinal Study. *PLoS One*. 2016; **11**: e0153924.
111. Wallensten J, Nager A, Asberg M, Borg K, Beser A, Wilczek A, et al. Leakage of astrocyte-derived extracellular vesicles in stress-induced exhaustion disorder: a cross-sectional study. *Scientific reports*. 2021; **11**: 2009-.
112. Wallensten J, Mobarrez F, Åsberg M, Borg K, Beser A, Wilczek A, et al. Isoforms of soluble vascular endothelial growth factor in stress-related mental disorders: a cross-sectional study. *Scientific reports*. 2021; **11**: 16693-.
113. Axelsson E, Lindsäter E, Ljótsson B, Andersson E, Hedman-Lagerlöf E. The 12-item Self-Report World Health Organization Disability Assessment Schedule (WHODAS) 2.0 Administered Via the Internet to Individuals With Anxiety and Stress Disorders: A Psychometric Investigation Based on Data From Two Clinical Trials. *JMIR Ment Health*. 2017; **4**: e58.
114. Beser A, Sorjonen K, Wahlberg K, Peterson U, Nygren A, Asberg M. Construction and evaluation of a self rating scale for stress-induced exhaustion disorder, the Karolinska Exhaustion Disorder Scale. *Scand J Psychol*. 2014; **55**: 72-82.
115. Lundgren-Nilsson A, Jonsdottir IH, Pallant J, Ahlborg G, Jr. Internal construct validity of the Shirom-Melamed Burnout Questionnaire (SMBQ). In: BMC Public Health: 2012.
116. Lundgren-Nilsson Å, Jonsdottir IH, Ahlborg JG, Tennant A. Construct validity of the Psychological General Well Being Index (PGWBI) in a sample of patients undergoing

treatment for stress-related exhaustion: a Rasch analysis. *Health and quality of life outcomes*. 2013; **11**: 2-.
117. Adevi AA, Lieberg M. Stress rehabilitation through garden therapy: A caregiver perspective on factors considered most essential to the recovery process. *Urban forestry & urban greening*. 2012; **11**: 51-8.
118. Adevi AA, Mårtensson F. Stress rehabilitation through garden therapy: The garden as a place in the recovery from stress. *Urban forestry & urban greening*. 2013; **12**: 230-7.
119. Eskilsson T, Fjellman-Wiklund A, Ek Malmer E, Stigsdotter Neely A, Malmberg Gavelin H, Slunga Jarvholm L, et al. Hopeful struggling for health: Experiences of participating in computerized cognitive training and aerobic training for persons with stress-related exhaustion disorder. *Scandinavian journal of psychology*. 2020; **61**: 361-8.
120. Finnes A, Ghaderi A, Dahl J, Nager A, Enebrink P. Randomized controlled trial of acceptance and commitment therapy and a workplace intervention for sickness absence due to mental disorders. *Journal of occupational health psychology*. 2019; **24**: 198-212.
121. Finnes A, Enebrink P, Sampaio F, Sorjonen K, Dahl J, Ghaderi A, et al. Cost-Effectiveness of Acceptance and Commitment Therapy and a Workplace Intervention for Employees on Sickness Absence due to Mental Disorders. *J Occup Environ Med*. 2017; **59**: 1211-20.
122. Karlson B, Jonsson P, Osterberg K. Long-term stability of return to work after a workplace-oriented intervention for patients on sick leave for burnout. *BMC Public Health*. 2014; **14**: 821.
123. Lindsater E, Axelsson E, Salomonsson S, Santoft F, Ejeby K, Ljotsson B, et al. Internet-Based Cognitive Behavioral Therapy for Chronic Stress: A Randomized Controlled Trial. *Psychotherapy and psychosomatics*. 2018; **87**: 296-305.
124. Lindsater E, Axelsson E, Salomonsson S, Santoft F, Ljotsson B, Akerstedt T, et al. Cost-Effectiveness of Therapist-Guided Internet-Based Cognitive Behavioral Therapy for Stress-Related Disorders: Secondary Analysis of a Randomized Controlled Trial. *Journal of medical Internet research*. 2019; **21**: e14675.
125. Lindsäter E, Axelsson E, Salomonsson S, Santoft F, Ljótsson B, Åkerstedt T, et al. The mediating role of insomnia severity in internet-based cognitive behavioral therapy for chronic stress: Secondary analysis of a randomized controlled trial. *Behaviour research and therapy*. 2021; **136**: 103782-.
126. Salomonsson S, Santoft F, Lindsäter E, Ejeby K, Ljotsson B, Ost LG, et al. Cognitive-behavioural therapy and return-to-work intervention for patients on sick leave due to common mental disorders: a randomised controlled trial. *Occup Environ Med*. 2017; **74**: 905-12.
127. Salomonsson S, Santoft F, Lindsater E, Ejeby K, Ingvar M, Ljotsson B, et al. Effects of cognitive behavioural therapy and return-to-work intervention for patients on sick leave due to stress-related disorders: Results from a randomized trial. *Scandinavian journal of psychology*. 2020; **61**: 281-9.
128. Santoft F, Salomonsson S, Hesser H, Lindsäter E, Ljótsson B, Lekander M, et al. Mediators of Change in Cognitive Behavior Therapy for Clinical Burnout. *Behavior Therapy*. 2019; **50**: 475-88.
129. Sonntag-Öström E, Nordin M, Slunga Järvholm L, Lundell Y, Brännström R, Dolling A. Can the boreal forest be used for rehabilitation and recovery from stress-related exhaustion? A pilot study. *Scandinavian journal of forest research*. 2011; **26**: 245-56.
130. Sonntag-Öström E, Stenlund T, Nordin M, Lundell Y, Ahlgren C, Fjellman-Wiklund A, et al. “Nature's effect on my mind”–Patients’ qualitative experiences of a forest-based rehabilitation programme. *Urban Forestry & Urban Greening*. 2015; **14**: 607-14.
131. Strömbäck M, Fjellman-Wiklund A, Keisu S, Sturesson M, Eskilsson T. Restoring confidence in return to work: A qualitative study of the experiences of persons with exhaustion disorder after a dialogue-based workplace intervention. *PloS one*. 2020; **15**: e0234897.
132. van de Leur JC, Buhrman M, Ahs F, Rozental A, Jansen GB. Standardized multimodal intervention for stress-induced exhaustion disorder: an open trial in a clinical setting. *BMC psychiatry*. 2020; **20**: 526-.
